# Mutations of the *Bacillus subtilis* YidC1 (SpoIIIJ) insertase alleviate stress associated with σ^M^-dependent membrane protein overproduction

**DOI:** 10.1371/journal.pgen.1008263

**Published:** 2019-10-18

**Authors:** Heng Zhao, Ankita J. Sachla, John D. Helmann

**Affiliations:** Department of Microbiology, Cornell University, Ithaca, NY, United States of America; Ohio State University, UNITED STATES

## Abstract

In *Bacillus subtilis*, the extracytoplasmic function σ factor σ^M^ regulates cell wall synthesis and is critical for intrinsic resistance to cell wall targeting antibiotics. The anti-σ factors YhdL and YhdK form a complex that restricts the basal activity of σ^M^, and the absence of YhdL leads to runaway expression of the σ^M^ regulon and cell death. Here, we report that this lethality can be suppressed by gain-of-function mutations in *yidC1* (*spoIIIJ)*, which encodes the major YidC membrane protein insertase in *B*. *subtilis*. *B*. *subtilis* PY79 YidC1 (SpoIIIJ) contains a single amino acid substitution in a functionally important hydrophilic groove (Q140K), and this allele suppresses the lethality of high σ^M^. Analysis of a library of YidC1 variants reveals that increased charge (+2 or +3) in the hydrophilic groove can compensate for high expression of the σ^M^ regulon. Derepression of the σ^M^ regulon induces secretion stress, oxidative stress and DNA damage responses, all of which can be alleviated by the YidC1^Q140K^ substitution. We further show that the fitness defect caused by high σ^M^ activity is exacerbated in the absence of the SecDF protein translocase or σ^M^-dependent induction of the Spx oxidative stress regulon. Conversely, cell growth is improved by mutation of specific σ^M^-dependent promoters controlling operons encoding integral membrane proteins. Collectively, these results reveal how the σ^M^ regulon has evolved to up-regulate membrane-localized complexes involved in cell wall synthesis, and to simultaneously counter the resulting stresses imposed by regulon induction.

## Author summary

Bacteria frequently produce antibiotics that inhibit the growth of competitors, and many naturally occurring antibiotics target cell wall synthesis. In *Bacillus subtilis*, the alternative σ factor σ^M^ is induced by cell wall antibiotics, and upregulates genes for peptidoglycan and cell envelope synthesis. However, dysregulation of the σ^M^ regulon, resulting from loss of the YhdL anti-σ^M^ protein, is lethal. We here identify charge variants of the YidC1 (SpoIIIJ) membrane protein insertase that suppress the lethal effects of high σ^M^ activity. Further analyses reveal that induction of the σ^M^ regulon leads to high level expression of membrane proteins that trigger envelope stress, and this stress is countered by specific genes in the σ^M^ regulon.

## Introduction

The ability of cells to adapt to changing conditions relies in large part on the expression of specific stress responses controlled by transcription regulators. The extracytoplasmic function (ECF) subfamily of σ factors are frequently involved in bacterial responses to stresses affecting the cell envelope [1, 2]. In *Bacillus subtilis*, there are seven ECF σ factors, with the σ^M^ regulon playing a particularly important role in modulating pathways involved in peptidoglycan synthesis, cell envelope stress responses, and intrinsic resistance to antibiotics [3]. Cells lacking σ^M^ (*sigM* null mutants) grow normally in unstressed conditions, but have greatly increased sensitivity to high salt and to cell wall active antibiotics, including ß-lactams and moenomycin [4–6].

Like many other ECF σ factors, the activity of σ^M^ is regulated by an anti-σ factor. For σ^M^, the anti-σ factor comprises two membrane-localized proteins encoded as part of the *sigM* operon, *sigM-yhdL-yhdK* [7, 8]. The major anti-σ factor is YhdL, a transmembrane protein that directly binds σ^M^ [9]. However, full YhdL activity requires a second transmembrane protein, YhdK. Although a lack of σ^M^ is well tolerated under unstressed conditions, the lack of the anti-σ^M^ factors leads to a runaway activation of the autoregulated *sigM* operon, and overexpression of the σ^M^ regulon [10]. A null mutation of *yhdK* leads to an ~100-fold elevation of σ^M^ activity, morphological abnormalities, and slow growth [10]. A null mutation in *yhdL* is lethal, but suppressors arise readily that have inactivated *sigM* and grow normally in unstressed conditions [4, 10]. These findings imply that high level expression of σ^M^ is toxic to cells, but the basis for this toxicity has not been defined.

Previously, we explored the basis for σ^M^ toxicity by selecting for suppression of *yhdL* lethality in a *sigM* merodiploid strain to reduce the frequency of suppressors that had inactivated σ^M^. These studies led to the recovery of mutations in *rpoB* and *rpoC*, encoding the ß and ß' subunits of RNA polymerase, that led to a reduction of σ^M^ activity sufficient to restore viability [10]. These mutations, which acted selectively on σ^M^, affected a region of core RNA polymerase involved in σ factor binding. In the course of these studies we also demonstrated that the toxicity from high σ^M^ could be alleviated by mutation of the autoregulatory promoter for the *sigM* operon, or by overexpression of the housekeeping σ factor, σ^A^ [10]. These results suggest that the lack of a functional anti-σ^M^ factor (*yhdL* null mutant) leads to runaway activation of the *sigM* operon and a high level of σ^M^ activity that is incompatible with growth. However, it is unclear whether σ^M^ toxicity results from a decrease in activity of the essential housekeeping σ^A^, overexpression of one or more σ^M^-regulated genes, or both.

To further explore the impact of overexpression of specific σ^M^-regulated genes on cell physiology we generated a library of strains in which specific σ^M^-dependent promoters (P_M_) are inactivated by point mutations. This approach, which removes σ^M^-dependent activation while leaving other promoters and regulatory inputs intact, is important since many σ^M^-regulated genes have multiple promoters and encode essential genes, including several involved in peptidoglycan synthesis and cell division [3]. In the course of developing this library we received a previously described strain with a mutation inactivating the P_M_ of *rodA*, encoding a SEDS family transglycosylase important for peptidoglycan synthesis [6]. We unexpectedly discovered that in this strain *yhdL* could be inactivated, and the same was true for the parent strain (*B*. *subtilis* PY79). These serendipitous observations led us to hypothesize that *B*. *subtilis* PY79 differs from other *B*. *subtilis* 168 strains in its ability to tolerate high level expression of the σ^M^ regulon.

Here, we report that the ability of *B*. *subtilis* PY79 to tolerate σ^M^ regulon overexpression results from a single amino acid substitution in the *yidC1 (spoIIIJ)* gene, which encodes the major YidC1 membrane insertase in *B*. *subtilis* [11, 12]. This finding motivated a detailed structure-function analysis of YidC1, which led to the discovery of mutations that increase the positive charge within the hydrophilic groove of YidC1 from +1 (wild-type in *B*.*subtilis* 168) to +2 or +3 (in specific combinations) and increase tolerance to overexpression of σ^M^-regulated membrane proteins. Moreover, high level activity of σ^M^ leads to induction of genes associated with secretion stress, oxidative stress, and DNA damage responses, and the σ^M^ regulon includes functions that help compensate for stresses associated with membrane protein overexpression.

## Results

### A single amino acid change in YidC1 (SpoIIIJ) is necessary and sufficient for tolerance of high σ^M^

**Fig 1:**
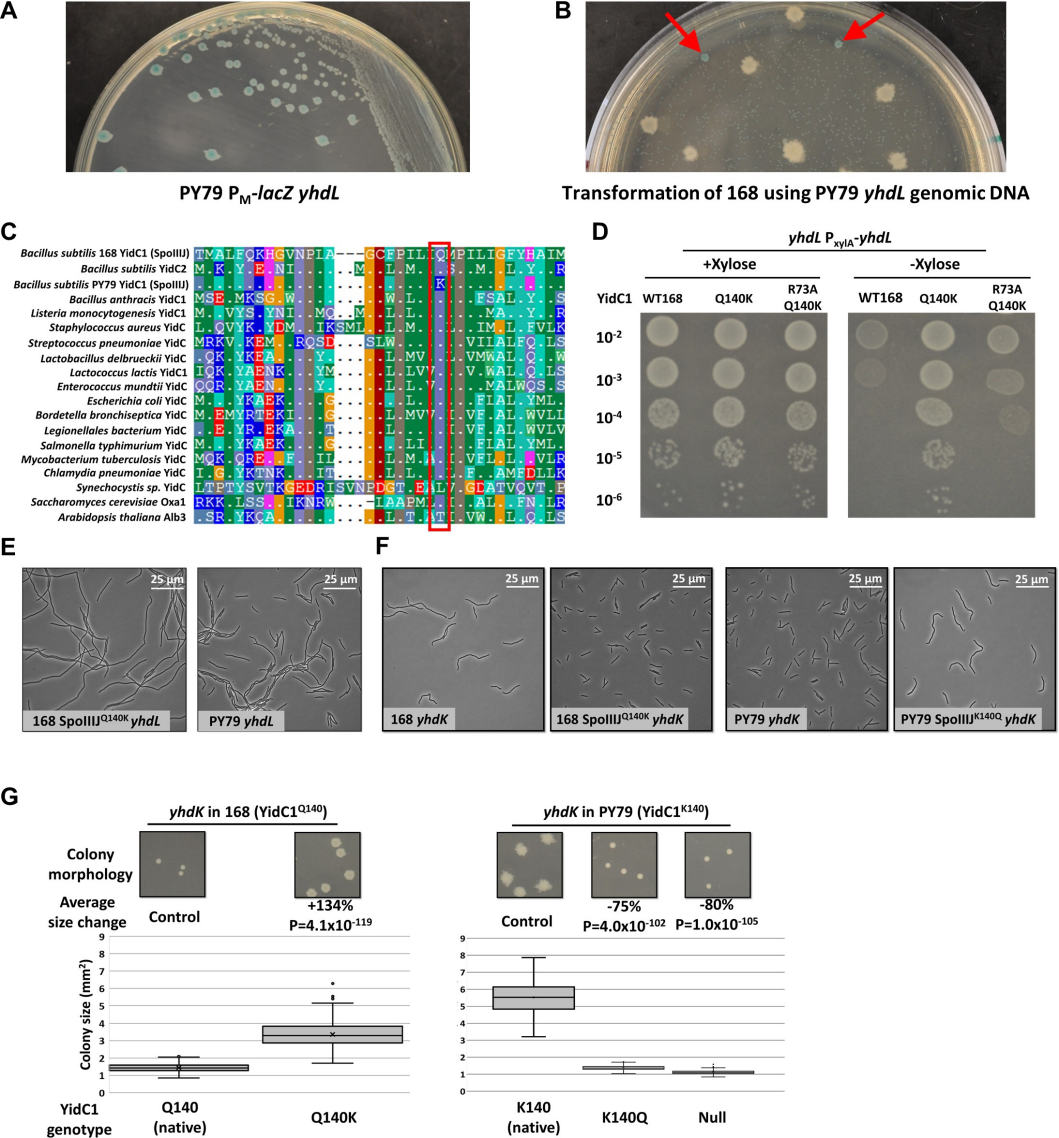
Identification of a YidC1 (SpoIIIJ) variant that is necessary and sufficient to abolish the essentiality of the anti-σ^M^ factor YhdL. A) Growth of PY79 P_M_-*lacZ yhdL* strain on LB plate with 60 μg/ml X-Gal. B) Transformation plate using 168 P_M_-*lacZ* as recipient and PY79 P_M_-*lacZ yhdL*::*kan* as DNA donor, with LB medium supplemented with kanamycin and X-Gal. Red arrows are pointed at intermediate sized blue colonies that were further analyzed by whole genome re-sequencing. C) Alignment of YidC homologs from bacterial and eukaryotic species. Red box highlights the aligned position corresponding to Q140 of *B*. *subtilis* YidC1. Residues identical to *B*. *subtilis* YidC1 are shown as “.” . D) Spot dilution of *yhdL* P_*xylA*_-*yhdL* depletion strain with different YidC1 alleles. WT168 has YidC1^Q140^ allele. E) Representative phase contrast microscopy image of *yhdL* mutants in different strain backgrounds. PY79 WT has a *yidC1*^K140^ allele. F) Representative phase contrast microscopy photo of *yhdK* mutants in different strain backgrounds. G) Colony morphology and size of *yhdK* mutants in different backgrounds. Colony size data were plotted using Box and Whisker chart and the bottom and top of the box are the first and third quartiles, the band inside the box is the second quartile (the median), and the X inside the box is the mean. Whiskers are one standard deviation above or below the mean. Outliers are shown as single dots. Average colony size change was calculated using formulation “change = (sample—control) / control x 100%”. P value was calculated using student’s t test, two tails, assuming unequal variances. Sample number n is at least 100 for each sample.

The anti-σ factors YhdL and YhdK regulate σ^M^, and the absence of *yhdL* is lethal in *B*. *subtilis* strain 168 due to toxic levels of σ^M^ [4, 10]. Since σ^M^ controls a large regulon, including many essential genes involved in cell wall synthesis [1, 3], we sought to construct a library of strains in which specific σ^M^-dependent promoters are inactivated by point mutations. One such promoter precedes *rodA*, which encodes a peptidoglycan transglycosylase [6]. We thus acquired a ΔP_M_-*rodA* strain (BAM1077 [6]), and tested whether *yhdL* is essential in that strain background, with its parent wild type strain PY79 as a control. Surprisingly, we found that *yhdL* is not essential in either PY79 strain. A *yhdL* mutant in PY79 exhibits reduced colony size compared to WT and high σ^M^ activity as indicated by the blue color on LB plates containing X-Gal (Fig 1A). This mutant is relatively stable, with the occasional appearance of suppressors that have a large white colony morphology (likely containing mutations in *sigM*). In contrast, introduction of a *yhdL* null mutation into other *B*. *subtilis* 168 strains results in tiny, pinpoint colonies that do not grow when re-streaked onto fresh plates, consistent with prior work [4, 10].

To identify the genetic differences in PY79 that confer tolerance of high σ^M^, we compared the genome between *B*. *subtilis* strain 168 and PY79. There are over a hundred single nucleotide polymorphisms (SNPs) between the 168 reference sequence and PY79, as well as four large deletions in the genome of PY79 (including the SPβ prophage), causing a reduction in size of 180 kb for the PY79 genome compared with 168 [13, 14]. To identify differences that might correlate with tolerance of high σ^M^ activity, we compared the sequences of genes encoding RNA polymerase subunits, and genes in the σ^M^ and Spx regulons (Spx is a transcription regulator that is regulated by σ^M^). However, no differences were noted in these 103 genes between the PY79 and 168 reference genomes (S1 Table).

Next, we used an unbiased, forward genetics approach to identity the mutation in PY79 that suppresses the lethality of a *yhdL* null mutation. We used genomic DNA from a PY79 *yhdL*::*kan* strain to transform 168 to kanamycin resistance, reasoning that the only viable transformants will likely also acquire the suppressing mutation. Because each competent cell of *B*. *subtilis* contains about 50 binding sites for DNA uptake, a competent cell can import multiple fragments of DNA during transformation in a process known as congression [15]. When a 168 strain containing a P_M_-*lacZ* reporter was transformed with chromosomal DNA from the viable PY79 *yhdL*::*kan* strain and selected on an LB plate supplemented with kanamycin and X-gal, we recovered numerous tiny blue colonies that did not grow when re-streaked onto fresh plates (consistent with the essentiality of YhdL in the 168 background), a few large white colonies (likely *sigM* mutants), and intermediate sized blue colonies (Fig 1B). The intermediate blue colonies grew to a similar size as a *yhdL* null mutant in PY79, consistent with acquisition of both the *yhdL*::*kan* allele and a second locus that suppresses the toxicity of high σ^M^. Whole genome sequencing was performed on these transformants and the reads were mapped to the reference genome of 168. Out of 15 sequenced 168 transformants, 14 contained the same SNP imported from PY79 that generates a missense mutation in *yidC1* (encoding YidC1^Q140K^) (S1A Fig, S2 Table). We therefore hypothesized that the *yidC1*^Q140K^ allele was the suppressor needed for cells to tolerate the *yhdL* mutation.

YidC1 belongs to the OXA1/ALB3/YidC family of protein insertases responsible for inserting membrane proteins into the lipid bilayer, independently or in association with the Sec secretion system [16–18]. *Escherichia coli* encodes one essential homolog of YidC, while some bacteria such as *B*. *subtilis* encode two homologs, YidC1 (SpoIIIJ) and YidC2 (YqjG) [18, 19]. The gene encoding YidC1 was originally named *spoIIIJ* because mutations at this locus lead to a block at stage III of sporulation [19, 20]. However, *spoIIIJ* is constitutively expressed and functional in vegetative cells. The expression of the paralog YidC2 is regulated by an upstream gene *mifM*, which monitors the total membrane protein insertase activity and only allows expression of YidC2 when MifM is not efficiently inserted into the membrane by YidC1 [21]. Both YidC1 and YidC2 can fulfill the essential function of YidC insertase, with YidC1 essential for sporulation [22] and YidC2 important for the development of competence (S2A Fig) [23]. Interestingly, an alignment of YidC homologs revealed that the Gln140 residue is highly conserved among bacteria, and only *B*. *subtilis* PY79 YidC1 contains Lys at this position (Fig 1C).

**Fig 2:**
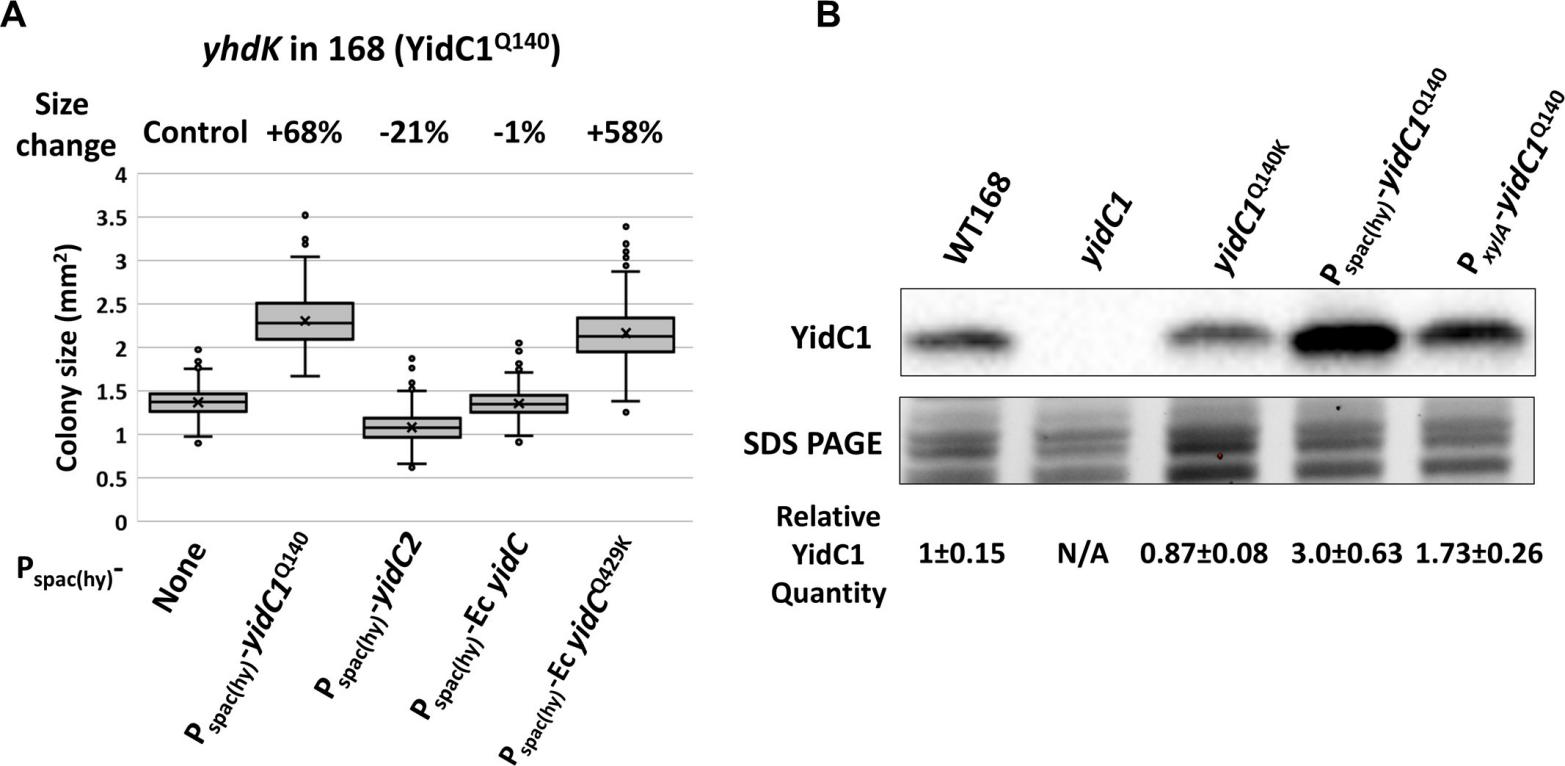
Overexpression of YidC1 increases tolerance of high σ^M^ activity. A) Colony size of *yhdK* mutants in 168 background with overexpression of YidC homologs using an IPTG inducible P_spac(hy)_ promoter. Cells were grown on LB plate supplemented with 1 mM IPTG for 24 hours at 37°C. Box and Whisker chart was plotted as in Fig 1G. B) Representative Western blot of YidC1 protein in different strain backgrounds. P_spac(hy)_ and P_*xylA*_ induced YidC1 strains contain the native YidC1 and were induced with 1 mM IPTG or 1% (w/v) xylose, respectively. Part of the SDS PAGE gel was shown as a loading control. Relative YidC1 quantity was calculated using band intensity values from three biological replicates and normalized using total lane intensity from the SDS PAGE gel. Data is presented as mean ± SEM.

To test if this YidC1 Gln to Lys variant (YidC1^Q140K^) is necessary and sufficient for tolerance of high σ^M^, we introduced the *yidC1*^Q140K^ mutation at the native locus of strain 168 using CRISPR-based mutagenesis, and found that *yhdL* was no longer essential (Fig 1D, S1B Fig). Conversely, changing the Lys140 into Gln in PY79 abolished the ability of PY79 to tolerate loss of *yhdL* (S1B Fig), suggesting that *yidC1*^Q140K^ is necessary and sufficient for tolerance of a *yhdL* deletion mutation. To test if the *yidC1*^K140^ allele is dominant over the *yidC1*^Q140^ allele, we constructed merodiploid strains expressing both alleles of *yidC1* (using a vector with xylose-inducible promoter P_*xylA*_ that integrates into *ganA* and when induced produces about 70% of the amount of the native protein; Fig 2B). Both merodiploid strains (168 expressing the PY79 allele, P_*xylA*_-*yidC1*^K140^, or PY79 expressing the 168 allele, P_*xylA*_- *yidC1*^Q140^) could still tolerate the loss of *yhdL* (S1B Fig). This dominance suggests that YidC1^Q140K^ leads to a gain of function that enables cells to tolerate high σ^M^ activity. Phase contrast microscopy revealed that a 168 *yidC1*^Q140K^ *yhdL* mutant had a similar but slightly more elongated cell morphology compared with a PY79 *yhdL* mutant (Fig 1E), confirming the major role of YidC1^Q140K^ in tolerance of a *yhdL* null mutation.

As a general strategy to monitor cell fitness, we compared the impact of *yidC1* alleles on morphology and colony size in the PY79 and 168 backgrounds for *yhdK* mutants. YhdK functions together with YhdL as an anti-σ complex, but unlike *yhdL* a *yhdK* null mutant is tolerated in 168 strains, although it does lead to an ~100-fold increase in σ^M^ regulon expression and severe growth defects [10]. Five independent trials with a 168 *yhdK* mutant, known to generate small colonies, revealed up to a ~10% change in average colony area. This variation likely results from small differences in the plating conditions. Although these small differences were in some cases judged to be statistically significant (based on P value in a t test, two tails, assuming unequal variances) (S1C Fig), this reflects the high sample number in each measurement (100–1000 colonies per measurement). Considering this level of variation between genetically identical strains, we only regard as significant those changes of >10% in colony size. Using this assay, we found that the small colony size, as well as the filamentous cell morphology of the 168 *yhdK* mutant, can be largely rescued by the *yidC1*^Q140K^ allele (Fig 1F and 1G). Conversely, a *yidC1*^K140Q^ mutation in PY79 *yhdK* converted the large colonies of the parent strain into the small round morphology of the 168 *yhdK* strain, with increased cell filamentation (Fig 1F and 1G). Deletion of *yidC1* in a PY79 *yhdK* mutant mimicked a *yidC1*^K140Q^ mutation (Fig 1G), likely because the cells now rely on the other YidC paralog, YidC2, which contains a glutamine residue in the equivalent position (Fig 1C) [19]. Overall, our results show that *yidC1*^Q140K^ mutation is necessary and sufficient for *B*. *subtilis* to tolerate high σ^M^ activity caused by the absence of the anti-σ factor YhdL or its partner protein YhdK.

### Overexpression of YidC1 increases tolerance of high σ^M^ activity

We hypothesized that the YidC1^Q140K^ protein may simply be more active or abundant in cells than the native protein. To test if increasing insertase activity is sufficient to alleviate toxicity associated with high σ^M^, we overexpressed the wild-type 168 YidC^Q140^ protein using a strong IPTG inducible promoter, P_spac(hy)_ [24]. Induction of the P_spac(hy)_-*yidC1*^Q140^ allele led to a three-fold increase in the amount of YidC1 protein compared with WT (Fig 2B), and increased the colony size of a *yhdK* mutant by 68% (Fig 2A). This increase is less than the effect of the *yidC1*^Q140K^ allele at the native locus (which increased *yhdK* colony size by 134%; Fig 1G), and consistently it only marginally increased the growth of a *yhdL* depletion strain under depletion condition (S2C Fig). The increase in the fitness of the *yhdK* mutant supports the hypothesis that higher insertase activity is beneficial for cells with elevated σ^M^ activity, but overexpression alone does not phenocopy the effect of the altered function allele.

We next tested whether overexpression of YidC2, the other YidC homolog in *Bacillus*, could benefit cells with high σ^M^ expression. Interestingly, when YidC2 was overexpressed from the P_spac(hy)_ promoter, the growth defect of either the *yhdK* mutant or the *yhdL* depletion strain was exacerbated (Fig 2A, S2C Fig). Furthermore, when the equivalent glutamine residue of YidC1^Q140^ was mutated to lysine, the YidC2^Q148K^ mutant protein was toxic when overexpressed in a *yhdK* mutant (S2B Fig). Thus, YidC2 is unable to compensate for YidC1 in alleviating stress associated with high σ^M^ activity, even when the corresponding Gln to Lys substitution is present. Similarly, overexpression of *E*. *coli* YidC did not provide any benefit to a *yhdK* or *yhdL* mutant (Fig 2A). However, when the equivalent Gln to Lys substitution was present, overexpression of the *E*. *coli* YidC^Q429K^ mutant was modestly beneficial, and colony size of the *yhdK* mutant increased by 58% (Fig 2A). These results suggest that different YidC homologs vary in their substrate preferences, and the Gln to Lys mutation may enhance the ability of the *B*. *subtilis* YidC1 and *E*. *coli* YidC insertases to facilitate membrane insertion of at least some σ^M^-dependent proteins.

### YidC1^Q140K^ increases the positive charge inside the substrate binding groove

**Fig 3:**
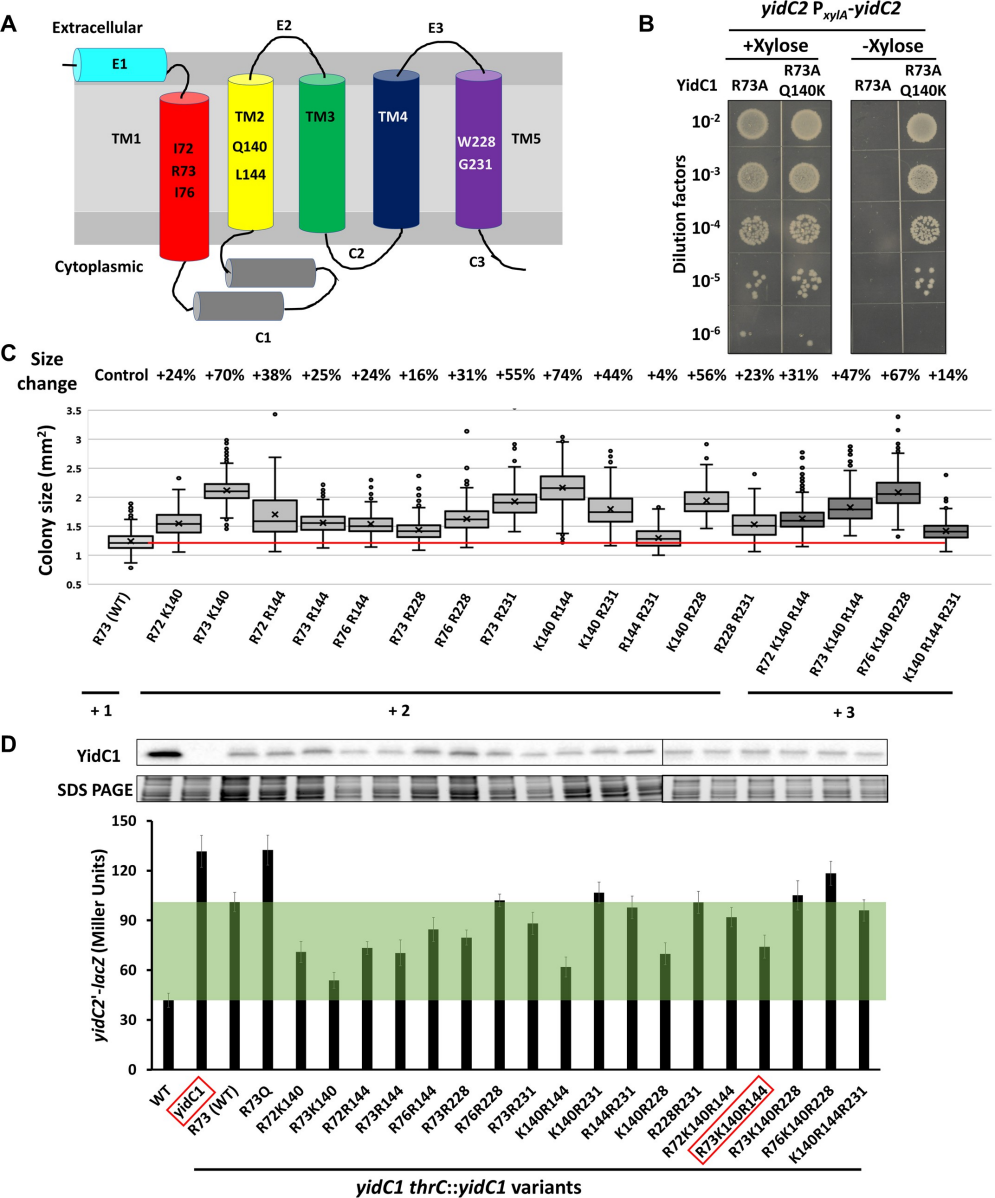
Effects of different charge variants inside the YidC1 hydrophilic groove. A) Schematic drawing showing the extracellular, transmembrane and cytoplasmic domains of YidC1. The native sequence in strain 168 of the seven residues for the positive charge library are shown. B) Spot dilution of *yidC2* P_*xylA*_-*yidC2* depletion strain with different YidC1 variants in strain 168. C) Colony size of *yhdK yidC1* mutants with different *yidC1* alleles integrated in the *thrC* site. Residues that provide positive charge are labelled, and the total positive charge provided by these seven variable positions is indicated. The red line shows the median of colony size with a WT YidC1 from strain 168. Box and Whisker chart was plotted as in Fig 1G. D) Top, representative Western blots of YidC1 protein in different strain backgrounds. Part of the SDS PAGE gels were shown as loading control. Bottom, activity of *yidC2*’-*lacZ* translational fusion in the same strain backgrounds of the western blots. “WT” has a native copy of *yidC1* while “*yidC1*” is a markerless deletion mutant. All other strains have the native *yidC1* deleted and contain variants of *yidC1* at *thrC* site. Among the seven variable positions, residues that provide positive charge(s) are labelled. YidC1 variants inside the red boxes cannot support growth of a *yidC2* P_*xylA*_-*yidC2* depletion strain in the absence of inducer xylose. Green shading highlights the difference between the activity of YidC1 at the native and *thrC* loci. Variants with *yidC2*’-*lacZ* activity inside the green shading exhibit increased MifM insertion activity compared with WT.

The structure of YidC2 from *Bacillus halodurans* revealed a positively charged hydrophilic groove formed by five transmembrane segments [16]. A positively charged residue in the substrate-binding groove is essential for the function of the insertase, as a R73A substitution in *B*. *subtilis* YidC1 completely abolished the essential function of YidC1 *in vivo*, while an R73K substitution retained function [16]. Although the positive charge is essential, the R73 residue is not, as the positive charge can be provided by substitution of any of six other residues inside the hydrophilic groove with Arg. These six positions include Ile72 and Ile76 in transmembrane region 1 (TM1), Gln140 and Leu144 in TM2, and Trp228 and Gly231 in TM5 [25] (Fig 3A, S3B Fig). Since both Arg and Lys are positively charged, we hypothesized that the key feature of the PY79 YidC1^K140^ protein is the +2 charge inside its substrate binding chamber. To test this hypothesis, we engineered a YidC1^R73AQ140K^ double substitution protein with a net charge of +1 in the groove, where R73 is functionally replaced by K140. Expression of this protein is sufficient to support viability, as judged in a strain with depletion of *yidC2* (Fig 3B), but is not sufficient to allow depletion of *yhdL* (Fig 1D). This supports the idea that the key effect of the Q140K substitution is to increase the positive charge in the substrate binding groove.

### Increased charge inside the substrate binding groove is key for tolerance of high σ^M^ activity

Since it is the increased positive charge from +1 to +2, rather than the Q140K substitution *per se*, that rescues cells from high σ^M^ toxicity, we next set out to test if other combinations of positively charged residues can also rescue cells from high σ^M^ activity. To this end, we generated a library of YidC1 variants with five of the six positions mentioned above substituted (or not) with Arg, as shown previously to support function in the absence of Arg73 [25], and Gln140 substituted (or not) with Lys, as seen in PY79. In addition, we mutated Arg73 to Gln (or not) (Fig 3A). This leads to 2^7^ = 128 possible charge combinations, ranging from 0 to a maximum of +7, with most containing a nominal positive charge of +2 to +5 in the substrate binding groove (S3A and S3B Fig). Note that, for the sake of simplicity, we assume that the K140 residue is positively charged since the epsilon-amino group of free Lys has a pKa of ~10.5, but the protonation state will likely depend on the local charge environment.

To identify YidC1 variants that can support growth of a *yhdL* depletion strain, we transformed a *yidC1* null *yhdL* depletion strain with a library of *yidC1* variants and selected for transformants that grew in the absence of *yhdL* induction. After sequencing and validation, we identified 103 *yidC1* mutants that supported growth in the absence of *yhdL* induction (S3 Table). Among them, 88 mutants contained a nominal double positive charge in 13 unique combinations (S3A Fig). Five of these 13 combinations include K140, including the combination present in the PY79 YidC1 protein: R73 K140. The remaining 15 mutants contained a nominal triple positive charge, with 4 unique combinations. Interestingly, all four of these variants contained K140, and in each case K140 was present together with a pair Arg residues that also supported growth with Q140. It is possible that the presence of nearby Arg residues lowers the pKa of K140, and the protonation state of this residue may also vary depending on the nature of the bound substrate. Among the 17 unique combinations of double and triple positive charges, none has more than one positive charge in TM1, whereas TM2 and TM5 can each harbor double positive charges (S3A Fig, S3 Table). We conclude that all 17 functional YidC1 variants have an effective charge of between +2 and +3 in the hydrophilic groove. This strongly suggests that a modest increase of charge inside this groove facilitates the recruitment and insertion of σ^M^-regulated proteins overproduced under YhdL depletion conditions, whereas a further increase may be detrimental to the activity or the stability of the insertase.

Each of these 17 YidC1 variants can alleviate the stress imposed by high σ^M^ activity and thereby support growth of the YhdL depletion strain (S4A Fig), and each increases the colony size of the *yhdK* mutant by up to 74% (Fig 3C). We also found 16 of these 17 variants can support growth of a YidC2 depletion strain (S4B Fig). Only the YidC1 R72 K140 R144 variant was unable to support cell growth under these conditions, suggesting that it is compromised in the ability to insert proteins essential for cell growth, despite its ability to modestly increase colony size of the *yhdK* mutant (31%; Fig 3C).

YidC1 inserts MifM into the membrane, which serves as a sensor of YidC1 function to regulate expression of *yidC2* [21, 26]. If the ability of YidC1 to insert MifM is reduced, then the *yidC2'-lacZ* fusion is induced. Therefore, we used a *yidC2’*-*lacZ* translational fusion reporter to measure the ability of each YidC1 variant to insert MifM. A WT 168 strain exhibited very low level of *yidC2*’-*lacZ* activity in the presence of native *yidC1* expression (~42 Miller Units (MU), Fig 3D), and deletion of *yidC1* increased the reporter activity by about 3-fold (~132 MU, Fig 3D). Complementation of a *yidC1* null mutant with the 168 version of *yidC1* (single positive charge at R73) at the *thrC* locus reduced the reporter activity to ~101 MU (Fig 3D). The lack of complete complementation may be caused by the location of the gene, as the native locus is close to the origin of the chromosome and thus has a higher copy number than *thrC* in fast growing cells. Indeed, under our growth condition (late exponential phase in LB at 37°C), less YidC1 protein was detected in the *thrC*::*spoIIIJ* complementation strain than the WT (Fig 3D).

Using this strain background, we found that most of the YidC1 variants with double positive charge appear to have higher MifM insertion ability as they exhibited lower *yidC2’*-*lacZ* activity than the WT protein. Interestingly, the three mutants with double positive charge that have the largest colony size in a *yhdK* mutant background (R73 K140, K140 R144, and K140 R228), also showed the lowest *yidC2’*-*lacZ* activity, consistent with the hypothesis that they have higher insertase activity for both MifM and for membrane proteins that contribute to toxicity in strains with high σ^M^-activity. Conversely, one mutant with a nominal triple positive charge (R76 K140 R228) exhibited a strong increase in colony size in the *yhdK* mutant background (Fig 3C), but appeared to have low MifM insertion activity (high *yidC2'-lacZ* expression; Fig 3D). Overall these results suggest that increasing the positive charge inside the substrate binding groove affects the efficiency of inserting membrane proteins, with several variants that appear to enhance the insertion efficiency for both MifM and σ^M^-regulated proteins, while also retaining the ability to insert essential membrane proteins.

### High σ^M^ activity causes a cascade of stresses that can be partially compensated by its regulon

YidC1 functions as a membrane insertase, by either independently inserting some single pass membrane proteins or by functioning as part of the Sec translocon to facilitate the folding of the translocated proteins [18, 27]. The finding that YidC1^Q140K^ can suppress growth defects caused by high σ^M^ leads us to reason that the toxicity of high σ^M^ is caused by overexpression of membrane proteins that overwhelm the secretion system, and that YidC1^Q140K^ suppresses this stress by functioning as a more efficient insertase/foldase or chaperone. In *B*. *subtilis*, membrane secretion stress is sensed by the CssRS (control of secretion stress regulator/sensor) two component system. In the presence of membrane secretion stress, caused either by overproduction of secreted proteins or high temperature, CssR upregulates expression of membrane proteases HtrA (high temperature requirement A) and HtrB to facilitate the re-folding or degradation of misfolded proteins [28, 29].

**Fig 4:**
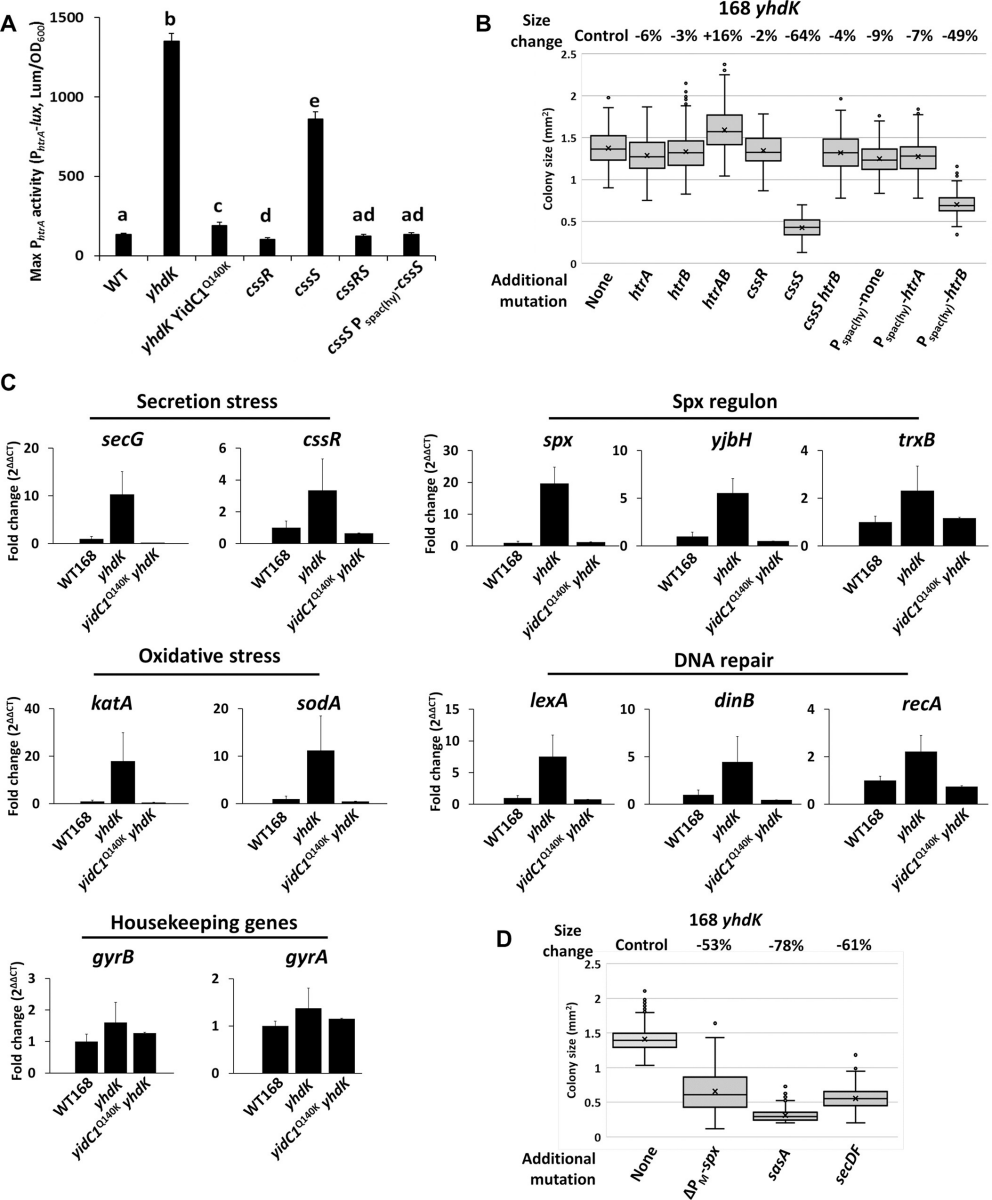
High σ^M^ activity causes a cascade of stresses that can be partially compensated by its own regulon. A) Maximum promoter activity of *htrA* measured using a P_*htrA*_-*lux* transcriptional fusion reporter in different strain backgrounds. Data is presented as mean ± SEM, and statistically significant different samples (Student’s t test, two-tailed P<0.05) are labelled with different letters. Sample number n is at least 4. B) Colony size of *yhdK* mutants in strain 168 with different additional mutations. Box and Whisker chart was plotted as in Fig 1G. C) Fold change of transcription levels of representative genes for stress response or housekeeping genes. Data is presented as mean ± SEM. Sample number n is at least 4. D) Colony size of *yhdK* mutants in strain 168 with different additional mutations. ΔP_M_-*spx* strain has the native *spx* deleted and an ectopic copy without the P_M_ promoter [43].

To test whether high σ^M^ causes secretion stress, we constructed a P_*htrA*_-*lux* reporter to monitor induction of the CssR regulon. Indeed, maximum P_*htrA*_ activity was increased ten-fold in a *yhdK* mutant, and this induction was greatly reduced in the presence of the *yidC1*^Q140K^ allele (Fig 4A, S5 Fig). As expected, deletion of the response regulator CssR abolished the induction of the regulon [29]. In contrast to a previous report [29], the absence of CssS increased transcription of P_*htrA*_-*lux* reporter by ~six-fold (Fig 4A, S5 Fig). This increased expression could be complemented by ectopic expression of CssS, and this depends on CssR as judged by analysis of a *cssS cssR* double deletion (Fig 4A). This suggests that CssS may act both as a sensor kinase to activate CssR under inducing conditions, and also as a phosphatase to deactivate CssR in the absence of induction, as also noted for sensor kinases in other two component systems [30].

To evaluate the role of the CssRS regulon in alleviating the stress associated with membrane protein overproduction, we constructed mutants lacking CssR-induced genes and used *yhdK* mutant colony size as a measure of fitness (Fig 4B). Surprisingly, we found that neither CssR-regulated membrane protease (HtrA and HtrB) is important for fitness of the *yhdK* mutant as judged by the effect of their deletion on colony size. Deletion of *cssS* reduced *yhdK* mutant colony size by over 60% (Fig 4B), and this reduction of colony size is reversed in a *cssS htrB* double mutant. This suggests that high HtrB activity is detrimental for the *yhdK* mutant. Consistently, when *htrB*, but not *htrA*, was overexpressed using the P_spac(hy)_ promoter, the colony size of the *yhdK* mutant was reduced by about 50% (Fig 4B). Deletion of these genes in the WT 168 background did not have much effect on the colony size (S6A Fig). These results suggest that the *yhdK* mutant is sensitive to the overproduction of membrane proteases. Single deletion of other proteases, including SipT, SipS, HtpX, PrsW and GlpG in a *yhdK* null mutant was also not detrimental, and in some cases led to a small beneficial effect in colony size (S6B Fig), again indicating that no single membrane protease is critical for *yhdK* mutant fitness. We speculate that under high σ^M^ conditions, the overproduced proteins may cause a backlog of membrane proteins that require a longer time to correctly insert and fold in the membrane. Some of these proteins may be essential for cell growth and are vulnerable to degradation by HtrB, and perhaps other proteases.

Misfolding of membrane proteins, as well as jamming the Sec translocon by hybrid proteins, leads to the generation of reactive oxygen species (ROS) and ultimately DNA damage [31, 32]. To test whether high σ^M^ also triggers a similar cascade of stresses, we performed quantitative RT-PCR to measure induction of representative stress genes. Indeed, the high σ^M^ activity in the *yhdK* null mutant was correlated with strong induction of genes involved in secretion stress (*secG*, *cssR*), oxidative stress (*katA*, *sodA*), and DNA repair (*lexA*, *dinB* and *recA*) (Fig 4C). The induction of these stress response genes was largely suppressed in the presence of the *yidC1*^Q140K^ allele, suggesting that their induction is a downstream effect resulting from inefficient insertion of membrane proteins (Fig 4C).

Interestingly, while many genes in the σ^M^ regulon are involved in the synthesis and maintenance of the cell wall, there are also genes involved in regulation of redox balance (*spx* and the Spx regulon), DNA repair and recombination (for example, *radA*, *radC* and *recU*), and ppGpp synthesis and stringent response (*sasA*) [3]. There is also a candidate σ^M^ promoter associated with the *secDF* operon [3]. This suggests that a subset of genes in the σ^M^ regulon are involved in compensating for stresses associated with upregulation of σ^M^. Indeed, deletion of *secDF*, *sasA* or the P_M_ of *spx* dramatically reduced the colony size of the *yhdK* mutant (Fig 4D), while there was very little effect noted in the WT background (S6C Fig). Thus, these genes seem to play an important role in cell fitness specifically under conditions of high σ^M^ expression. Deleting single genes inside the Spx regulon in the *yhdK* mutant did not lead to noticeable reduction of colony size (S6D Fig), suggesting functional redundancy within the Spx regulon.

**Fig 5:**
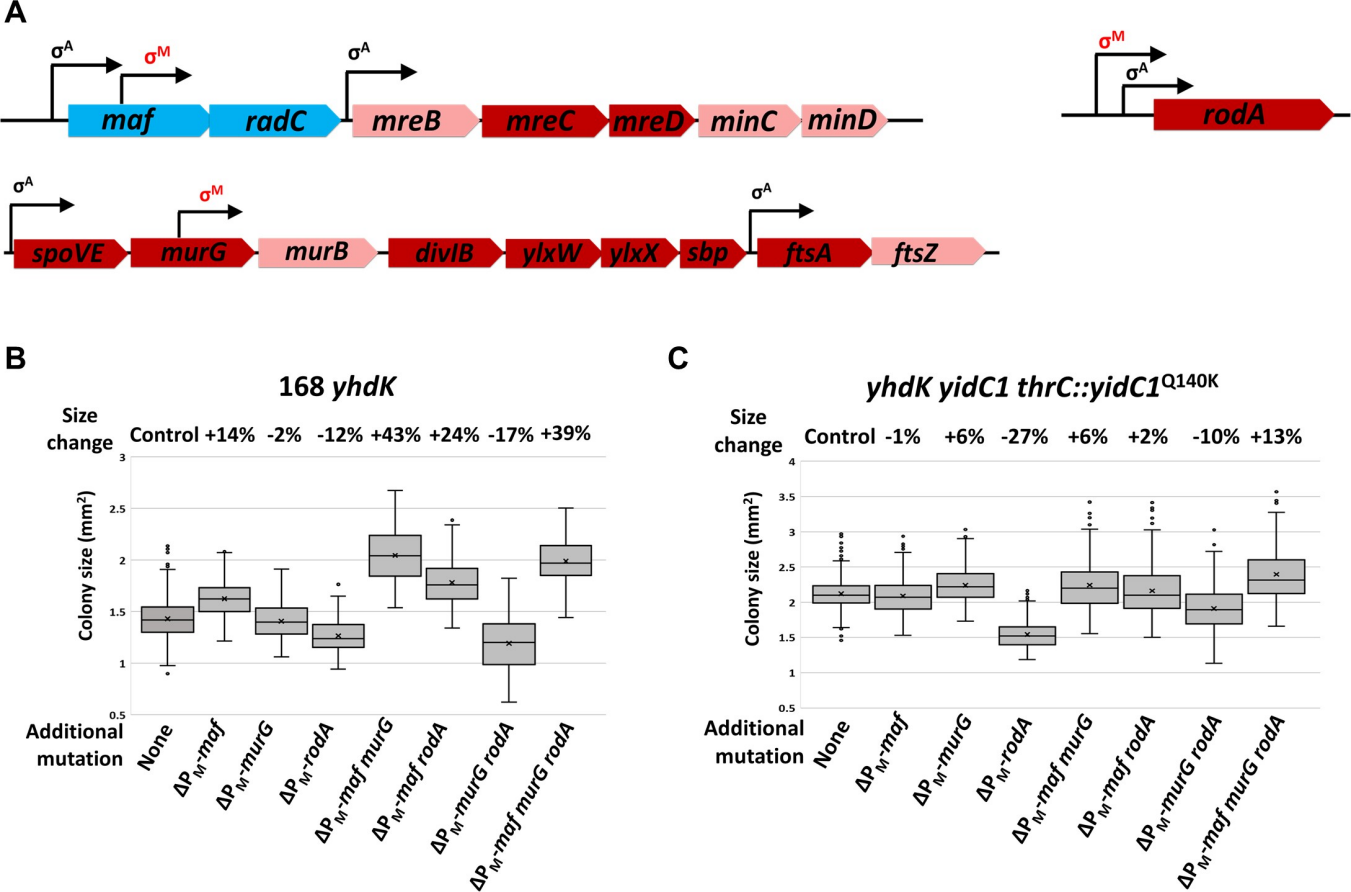
Preventing induction of specific σ^M^-dependent membrane proteins alleviates stresses caused by high σ^M^ activity. A) Schematic drawing of the *maf* and *murG* operons and the *rodA* gene. σ^A^ and σ^M^ controlled promoters are shown with arrows. Cytoplasmic proteins are labelled in blue boxes, peripheral membrane proteins in pink boxes, and integral membrane proteins in red boxes. B,C) Colony size of *yhdK* mutants with different P_M_ promoter(s) mutated in (B) WT strain 168 or (C) strain 168 with the native *yidC1* deleted and a *yidC1*^Q140K^ allele integrated at the *thrC* site. Box and Whisker chart was plotted as in Fig 1G.

Among the 69 genes currently assigned to the σ^M^ regulon according to SubtiWiki [33], 38 code for membrane-associated or secreted proteins (S4 Table). To demonstrate the burden these membrane proteins may cause when overexpressed under high σ^M^ condition, we mutated the σ^M^-dependent promoters (P_M_) for genes important for the elongasome and the divisome and tested the consequence on fitness measured by colony size of the *yhdK* mutant. We focused on a P_M_ inside the *maf* gene that transcribes the *radC*-*mreB*-*mreC*-*mreD*-*minC*-*minD* operon, a P_M_ upstream of *rodA* gene, and a P_M_ inside *murG* that contributes to transcription of the *murB*-*divIB*-*ylxW*-*ylxX*-*sbp*-*ftsA*-*ftsZ* operon (Fig 5A). Mutation of these three promoters individually or in combination had little effect on the colony size of the WT strain (S6E Fig), since these genes are also expressed from σ^A^-dependent promoters. In a *yhdK* mutant, however, mutation of P_M_(*maf*) and P_M_(*murG*) led to an additive increase of colony size, while mutation of P_M_(*rodA*) led to a small detrimental effect (Fig 5B). We conclude that overexpression of genes downstream of P_M_(*maf*) and P_M_(*murG*) may overwhelm the membrane-protein insertion pathway, and thereby contribute to a net negative effect on cell fitness. Consistent with this idea, in cells expressing YidC1^Q140K^, the beneficial effect of mutating P_M_(*maf*) and P_M_(*murG*) was largely abolished (Fig 5C).

## Discussion

The YidC membrane protein insertase is the bacterial representative of the YidC/Oxa1/Alb3 protein family of evolutionary conserved integral membrane proteins [18, 27]. In *E*. *coli*, YidC is required for the assembly of one or more essential protein complexes that reside in the inner membrane, including subunits of the energy generating F_1_F_O_ ATPase and NADH dehydrogenase I [34]. Although YidC can function in concert with the SecYEG translocon as a foldase/chaperone, YidC can also function independently for the insertion of small membrane proteins with one or two transmembrane segments [35].

*Bacillus subtilis* encodes two YidC paralogs, YidC1 (SpoIIIJ) and YidC2 (formerly YqjG). Either protein can support growth of *B*. *subtilis*, but a double mutant is inviable. Under most conditions YidC1 is the functional YidC homolog and is responsible for insertion of transmembrane proteins, including the F_1_F_O_ ATPase [19]. MifM, a membrane protein encoded upstream of *yidC2*, is a monitoring substrate of YidC1 and serves as a sensor for YidC1 activity [21]. In the absence of YidC1 activity, a prolonged arrest during MifM translation occurs that alters the *mifM-yidC2* mRNA structure to expose the ribosome-binding site of *yidC2*, thereby activated YidC2 expression [26, 36]. Although either YidC1 or YidC2 can support growth, they appear to differ in the efficacy of inserting specific proteins: cells lacking YidC1 are impaired in sporulation [20], whereas those lacking YidC2 have decreased competence [23]. The relationship between YidC protein sequence and the selection of specific client proteins is poorly understood. In cells depleted for both YidC1 and YidC2 there was a substantial upregulation in expression of the Clp protease system and the LiaIH membrane-stress proteins that are regulated by the LiaRS two-component system [23]. This suggests that impairment of membrane protein insertion leads to an accumulation of misfolded proteins and disruption of the cell membrane.

One critical feature required for YidC function, as first visualized in the structure of the *B*. *halodurans* YidC protein [16], is a hydrophilic groove postulated to interact transiently with transmembrane segments of nascent integral membrane proteins. In YidC proteins the first transmembrane region (TM1) contains a conserved Arg residue that is essential for function in many, but not all, YidC orthologs [37]. This positively charged residue is postulated to form a salt-bridge with single-pass transmembrane client proteins with acidic residues in their amino-terminal region [16]. Remarkably, the function of this conserved Arg residue (R73 in *Bacillus* YidC1) can be replaced by Arg residues introduced at any of six other positions in transmembrane segments of *B*. *halodurans* YidC [25].

In this work, we have described an unusual variant of YidC1 found in *B*. *subtilis* strain PY79 which contains the conserved R73 residue and additionally a second positively charged residue (K140) at a position that can functionally replace R73. This variant YidC1 protein (YidC1^Q140K^) is necessary and sufficient for *B*. *subtilis* to survive high level expression of the σ^M^ regulon. By screening a library of YidC1 variants, we revealed that all YidC1 proteins that enable cells to tolerate loss of *yhdL* contain at least two, and in some cases three, positively charged residues in this groove. We postulated that these YidC1 variants may be more capable of accommodating the increased expression of membrane proteins under σ^M^ control. Support for this hypothesis derives from experiments in which the deletion of individual σ^M^-dependent promoters that control operons encoding multiple integral membrane proteins was found to improve fitness of strains with high σ^M^ activity. Thus we reason that the relevant feature of YidC1^Q140K^ is the presence of increased positive charge in the presumed substrate-binding groove, which probably results in an increased insertase/foldase activity for specific σ^M^-regulated membrane proteins.

A similar selective pressure may also account for the reproducible recovery of a missense mutation encoding a YidC1 Trp138Arg substitution (YidC1_Sm_^W138R^) in a *Streptococcus mutans* strain lacking YidC2 [38]. In this case, the lack of YidC2 leads to a variety of phenotypes including poor growth, acid and osmotic stress sensitivity, and a lack of competence. The YidC1_Sm_^W138R^ protein retains the positive charge of Arg66 (equivalent to Arg73 in *B*. *subtilis* YidC1) and additionally has a second positive charge contributed by Arg138 (equivalent to Ile145 in *B*. *subtilis* YidC1 and adjacent to position 144, where an Arg substitution can functionally replace Arg73 [25]). A similar functional variant (R73, R144) was recovered three times from our *B*. *subtilis* YidC1 library (#40, 65, and 74; S3 Table).

Our results have provided further insight into the nature of the lethality associated with a *yhdL* deletion that unleashes high level σ^M^ activity [10]. In strains with elevated σ^M^ activity there is an increased flux of proteins targeted to the membrane and a subset of these may be inefficiently inserted by the native YidC1 protein. This can result in a jamming of YidC1-dependent protein translocation and a disruption of membrane function. The downstream sequelae associated with this disruption include misfolding of proteins and induction of the secretion stress response (CssR), as well as induction of genes associated with oxidative stress and DNA damage responses. These types of stresses may explain, in part, the inclusion of appropriate compensatory functions (including Spx, some DNA repair functions, and SecDF) as part of the σ^M^ regulon. It is presently unclear why the *yidC1*^*Q140K*^ allele arose in the PY79 strain, or what conditions may have led to its selection. However, this strain was derived from strains treated with chemical mutagens to cure the endogenous prophage SPß [14] and this or other selection conditions may have contributed to emergence of this mutation. Further studies will be required to better understand how variations in YidC structure can fine-tune the substrate selectivity of this essential membrane protein insertase, and the stresses that arise when this system is challenged by the induction of highly expressed membrane proteins.

## Materials and methods

### Strains, plasmids and growth condition

All strains used in this work are listed in S5 Table, and all DNA primers are listed in S6 Table. Bacteria were routinely grown in liquid lysogeny broth (LB) with vigorous shaking, or on plates (1.5% agar; Difco) at 37°C unless otherwise stated. LB medium contains 10 g tryptone, 5 g yeast extract, and 5 g NaCl per liter. Plasmids were constructed using standard methods [39], and amplified in *E*. *coli* DH5α or TG1 before transforming into *B*. *subtilis*. For selection of transformants, 100 μg ml^-1^ ampicillin or 30 μg ml^-1^ kanamycin was used for *E*. *coli*. Antibiotics used for selection of *B*. *subtilis* transformants include: kanamycin 15 μg ml^-1^, spectinomycin 100 μg ml^-1^, macrolide-lincosamide-streptogramin B (MLS, contains 1 μg ml^-1^ erythromycin and 25 μg ml^-1^ lincomycin), and chloramphenicol 10 μg ml^-1^. To reduce the likelihood of suppressors in *yhdK* mutants, fresh transformants were isolated and grown for assays using *yhdK* mutant strains. For spot dilution assays, cells were first grown in liquid culture at 37°C with shaking to mid-exponential phase (OD_600_ ~0.3–0.4), washed twice in LB medium without inducer, then serial diluted in LB medium without inducer. 10 μl of each diluted culture was then spotted onto plates and allowed to dry before incubation at 37°C for 12–24 hours.

### Genetic techniques

Chromosomal and plasmid DNA transformation was performed as previously stated [40]. The pPL82 plasmid-based P_spac(hy)_ overexpression constructs were sequencing confirmed before linearized and integrated into the *amyE* locus [24]. The pAX01 plasmid-based P_*xylA*_ overexpression constructs were sequencing confirmed before linearized and integrated into the *ganA* locus [41]. The *ganA*::P_*xylA*_-*yhdL*-*cat* and *thrC*::P_M_-*spoVG*-*lacZ*-*spec* constructs were made with LFH PCR to avoid antibiotic marker conflicts [10]. Markerless in-frame deletion mutants were constructed from BKE or BKK strains as described [42]. Briefly, BKE or BKK strains were acquired from the *Bacillus* Genetics Stock Center (http://www.bgsc.org), chromosomal DNA was extracted, and the mutation containing an *erm*^R^ (for BKE strains) or *kan*^R^ (for BKK strains) cassette was transformed into our WT 168 strain. The antibiotic cassette was subsequently removed by introduction of the Cre recombinase carried on plasmid pDR244, which was later cured by growing at the non-permissive temperature of 42°C. Gene deletions were confirmed by PCR screening using flanking primers. Unless otherwise described, all PCR products were generated using *B*. *subtilis* 168 strain chromosomal DNA as template. DNA fragments used for gene over-expression were verified by sequencing. Null mutant constructions were verified by PCR.

Mutations to selectively inactivate σ^M^-dependent promoters were generated by either promoter deletion (*rodA*) or by inactivating point mutations (*murG*, *maf*, *spx*). To inactivate P_M_(*rodA*), a 91 bp region containing the P_M_ (located upstream of a σ^A^-dependent promoter) was deleted using CRISPR. The resulting deletion had a junction sequence of: CACATTATCGC/TTTCGTGTAGC. The point mutations inactivating *murG* and *maf* both changed the -10 region sequence from consensus, CGTC, to TGTT. The P_M_* mutation inactivating the promoter regulating the *yjbC-spx* operon is a 3 bp substitution changing the -10 region from consensus, CGTC, to AAGT, as previously described [43].

Mutations of *yidC1* at native locus, as well as ΔP_M_-*rodA* were constructed using a clustered regularly interspaced short palindromic repeats (CRISPR)-based mutagenesis method as previously described [10]. Briefly, possible protospacer adjacent motifs (PAM) site, which is NGG for *Streptococcus pyogenes* Cas9, was identified and off-target sites were checked against *B*. *subtilis* genome using BLAST. If no off-target site was identified, the PAM site was chosen and 20 bps upstream of the site were used as sgRNA and cloned into vector pJOE8999 [44]. The repair template was generated by joining two or more PCR products, with intended mutation (with additional mutation to abolish gRNA recognition if necessary) introduced by PCR primers, and cloned into the pJOE8999-sgRNA vector. DNA sequence for amino acid substitution was chosen according to the preferred codons of *B*. *subtilis* [45]. The pJOE8999 derivative containing both the sgRNA and repair template was then cloned into competent cells of *E*. *coli* strain TG1 to produce concatemer plasmids, which were transformed into *B*. *subtilis* at 30°C. Transformants were then grown at 42°C to cure the plasmid, and intended mutations were confirmed by sequencing. ΔP_M_-*rodA* was constructed with repair template amplified using primers 7426, 7427, 7428 and 7429, and the gRNA constructed using 7430 and 7431. YidC1^Q140K^ mutation in 168 was constructed with repair template amplified using primers 7868, 7869, 7870 and 7871, and the gRNA constructed using 7866 and 7867. YidC1^K140Q^ mutation in PY79 was constructed with repair template amplified using primers 7866, 8247, 8246 and 7871, and the gRNA constructed using 8248 and 8249. YidC1^R73A^ mutation was constructed with repair template amplified using strain 168 genomic DNA as template with primers 7868, 8280, 8281 and 7871, and the gRNA constructed using 8278 and 8279. YidC1^R73AQ140K^ mutation was constructed with the same primer sets for YidC1^R73A^ mutation, expect the repair template was amplified using strain PY79 genomic DNA. All constructs were sequencing confirmed.

A YidC1 library of varying positive charge composition was constructed using degenerate primers and LFH PCR. Four DNA fragments were amplified and joined using LFH PCR. The fragments include one with part of gene *hom* and *thrC* (amplified with primers 8766 and 8767), one with the *yidC1* gene from 168 (amplified with primers 8768 and 8681, containing the native promoter and ribosomal binding site), one with a *spec*^R^ cassette (amplified with primers 8682 and 8769), and one with part of *thrC* and full length of *thrB* (amplified with primers 8770 and 8771). The joined PCR product was first transformed into strain 168 to generate HB23976. Then using genomic DNA of HB23976 as template, four new DNA fragments were amplified using degenerate primer pairs 8766 and 8689, 8686 and 8690, 8687 and 8691, and 8688 and 8771. These fragments were joined together using LFH PCR and the joined PCR product (more than 10 μg DNA) was expected to contain all 128 possible combinations of YidC1 charge from 0 to +7. The PCR product library was used to transform strain 168, and more than ten-thousand transformants (from 20 plates, each contains more than 500 colonies) were pooled together to extract genomic DNA to form a virtually equivalent DNA library of the *yidC1* variants. This genomic DNA library provides high transformation efficiency and was used to transform a *yhdL* depletion strain (HB23953) and transformants were selected on LB plate supplemented with X-gal but no xylose for *yhdL* induction. More than 200 transformants were re-streaked onto fresh LB plate with X-gal but no xylose to confirm robust growth under high σ^M^ condition, and 103 of them were Sanger sequenced for the *thrC*-*yidC1*-*spec* region to identify the *yidC1* variants.

### Whole genome re-sequencing and sequence analysis

Chromosomal DNA of suppressor strains was extracted using Qiagen DNeasy Blood & Tissue Kit. DNA was then sent to Cornell University Institute of Biotechnology for sequencing using Illumina HiSeq2500 with Single-end 100 bp reads. Sequencing results were analyzed using CLC workbench version 8.5.1 and mapped to the genome of strain 168 (reference accession number NC_000964.3). Note that our working stock of *B*. *subtilis* 168 has 21 SNPs compared to the cited reference sequence, and these common SNPs were not considered, and only newly introduced SNPs from the PY79 strain were tabulated (S2 Table). The unmapped reads were de novo assembled and contigs larger than 1 kb were aligned using the BLAST algorithm against the genome of strain PY79 (reference accession number NC_022898.1). Single nucleotide variants (SNVs) were detected using default settings, and gene deletions larger than 300 bps were identified by manually scanning regions of low coverage.

### Colony size measurement

Colony size was measured using Fiji Image J [46]. Briefly, bacterial cells were grown in liquid LB medium at 37°C with vigorous shaking to mid-exponential phase (OD_600_~0.3–0.4), then serial diluted to desired concentrations. Diluted cells were plated onto fresh LB plates (15 ml medium per plate, the diameter of the plate is 10 cm and the height 15 mm, VWR, US, Catalog number 25384–342), and multiple dilutions were used. Plates were incubated at 37°C for 24 hours. Plates containing less than 100 separate single colonies were used for size measurement, because this number of colonies per plate ensures sufficient sample size and does not cause reduced colony size due to crowdedness and nutrient limitation. Pictures of plates were taken with a ruler as a length reference, and colony size was measured using Fiji Image J per software’s instruction. For each strain, at least 100 colonies were measured, and box and whisker plots were used.

### Luciferase reporter construction and measurement

Luciferase reporter construction and measurement was performed as previous described [47]. The luciferase reporters were constructed by inserting the tested promoters into the multicloning site of pBS3C*lux* [48]. The promoter P_*htrA*_ was amplified using primers 8403 and 8404. The original chloramphenicol resistant cassette was replaced by an erythromycin resistance cassette when necessary to avoid antibiotic resistance conflicts. For luciferase measurements, 1 μl of exponentially growing cells were inoculated into 99 μl of fresh medium in a 96 well plate, incubated at 37°C with shaking using a SpectraMax i3x or a Synergy H1 (BioTek Instruments, Inc. VT) plate reader, and OD_600_ and luminescence were measured every 12 min. The data was analyzed using SoftMax Pro 7.0 software. Promoter activity was normalized by dividing the relative light units (RLU) by OD_600_.

### Phase contrast microscopy

Cells were grown in liquid LB medium to exponential phase (OD_600_ ~0.4) and loaded on saline (0.90% NaCl, w/v) agarose pads (0.8% final concentration) on a glass slide. Phase contrast images were taken using a Leica DMi8 microscope equipped with a 100x immersion objective and Leica Application Suite X software.

### Western blot

Western blot was performed as described previously [43]. Briefly, cells were grown in 5 ml LB medium in a 20 ml test tube a 37°C with vigorous shaking. Inducer IPTG or xylose were added when required by the construct to induce YidC1. After reaching exponential phase (OD_600_~0.3–0.4), 1 ml cells were pelleted by centrifugation at 4°C, resuspended in 100 μl pre-chilled buffer (containing 25 μl 4X Laemmli Sample buffer (Bio-Rad, USA), 10 μl 1M DTT, 65 μl H_2_O) and kept on ice. Cells were then lysed and crude cell lysate was loaded to a 4–20% SDS-PAGE stain-free gel for electrophoresis. The gel was visualized using ImageLab with stain-free gel protocol. Proteins were then transferred onto a PVDF membrane using the TransBlot Turbo Transfer System (Bio-Rad, USA), and immune blotting was performed using anti-YidC1 antiserum. The blot was visualized using the Clarity Western ECL substrate (Bio-Rad) and ImageLab software. Band intensity was calculated using the ImageLab software and normalized using total protein amount according to SDS-PAGE gel image.

### β-galactosidase assay for *yidC2’*-*lacZ*

Bacterial cells containing the *yidC2’*-*lacZ* translational fusion were grown to late exponential phase (OD_600_ 0.6–0.8) in 96-well plates with 200 μl LB medium per well at 37°C with vigorous shaking. Cells were pelleted by centrifugation, resuspended in Z buffer supplemented with DTT (dithiothreitol, 400 nM final concentration), and lysed by lysozyme. OD_600_ was measured before lysozyme treatment. After lysis, ONPG (ortho-nitrophenyl-β-galactoside) was added and OD_420_ and OD_550_ were measured every 2 minutes. Product accumulation was calculated using formula product = 1000×[OD_420_-(1.75×OD_550_)] and plotted against time. The slope of the linear part of the product accumulation curve was calculated using Excel and Miller Units (MU) were calculated using formula MU = Slope/OD_600_/V, where V is the volume of cells used for the reaction (200 μl).

### RNA extraction and qRT-PCR

Cells were grown to mid-exponential phase (OD_600_ ~0.4) and RNA was extracted using RNeasy Mini Kit (Qiagen). The extracted was then treated with DNase I (Invitrogen) and the quality of RNA was checked with electrophoresis. RNA was then reverse transcribed into cDNA using High-Capacity cDNA Reverse Transcription Kit (Applied Biosystems). Quantitative real-time PCR (qRT-PCR) was performed using SYBR Green (BioRad) and the *topA* gene was used for reference of data normalization.

## Supporting information

S1 Fig A) Map of SNPs from strain PY79 to 168 and distribution of SNPs contained in each congression suppressors. Genome coordinates were based on the 168 reference genome with NCBI accession number NC_000964.3. B) Transformation plates of *yhdL*::*kan* allele transformed into different strain backgrounds, selected on LB plates supplemented with kanamycin, X-gal and 1% xylose. C) Variation of *yhdK* colony size measurement between trials on different days with different batches of LB plates. P value was calculated using Student’s t test, and percentage changes of average colony size were shown.
(TIF)

S2 Fig A) Venn diagram of function overlap and distinction of YidC1 and YidC2 of *Bacillus subtilis*. B) Streaking of *yhdK* P_spac(hy)_-*yidC2*^Q148K^ on plates of LB or LB supplemented with 1 mM IPTG (final concentration). C) Spot dilution of *yhdL* depletion strains with P_spac(hy)_ based overexpression of different YidC homologs, on LB plates supplemented with a final concentration of 1% xylose (+Xylose), 1 mM IPTG (+IPTG) or nothing (None).
(TIF)

S3 Fig A) Distribution of 128 possible charge variants of YidC1 in the tested library. Theoretically, the majority of YidC1 variants in the input library have a charge of +2 to +5 in the hydrophilic groove, while experimental data from 103 samples suggests that the ones capable of providing high σ^M^ tolerance contain a charge of +2 or +3. Among the 103 samples with charge of +2 or +3, the positive charge can be located in one, two or three transmembrane segments, with the exception that no sample contains more than one positive charge in TM1 alone. B) Crystal structure of YidC from *Bacillus halodurans* (PDB ID 3WO6), showing the seven variable amino acid providing positive charges in the hydrophilic substrate binding chamber of the enzyme. Gly231 is not visible due to the lack of side chain of this residue. TM1-5, transmembrane region 1–5; E1, extracytoplasmic region 1; C1, cytoplasmic region 1.This figure was generated using UCSF Chimera 1.13[49].
(TIF)

S4 Fig A) *yhdL* depletion strains with YidC1 variants were streaked on LB plates with or without xylose inducer for *yhdL*. The positive charge of each variant was labelled next to the streaking, with a negative control “None” meaning no *yidC1* variant at *thrC* locus (weak growth due to the depletion conditions, and cannot be restreaked), and a positive control “YidC1_PY79_” meaning the native YidC1 mutated into the PY79 Q140K version (HB23719). B) Spot dilution of YidC depletion strains with *yidC1* variants at *thrC* locus. The depletion strain has its native *yidC1* and *yidC2* deleted, and a xylose inducible copy of P_*xylA*_-*yidC2*. Diluted cultures were spotted on LB without xylose (-Xyl) or with 1% final concentration of xylose (+Xyl). The negative control (None) has no *yidC1* at *thrC* locus, while the positive control has the 168 version of *yidC1* that contains a single positive charge at R73. Some YidC1 variants exhibited reduced growth ability, and the variant containing R72 K140 R144 failed to grow, although the emergence of suppressors was noted.
(TIF)

S5 Fig A) Growth curves of WT strain 168, *yhdK* null, and *yhdK* with YidC1^Q140K^ single amino acid substitution. B) P_*htrA*_ activity of strains in panel A during growth. C) Growth curves of WT strain 168, *cssR* null, *cssS* null, *cssRS* double null, and *cssS* null with an ectopic IPTG inducible copy under induced or uninduced conditions. D) P_*htrA*_ activity of strains in panel C during growth. The OD_600_ and luminescence were measured every 12 minutes. At least four biological replicates were used for each strain, and the results are shown as mean ± SEM.
(TIF)

S6 Fig Colony size of various *B*. *subtilis* strains compared to the relevant control strain. A) WT *B*. *subtilis* 168 vs. strains with the indicated mutations. B) *B*. *subtilis yhdK* null mutant vs. strains additionally mutant for the indicated gene. C) WT *B*. *subtilis* 168 vs. strains lacking either the σ^M^-regulated promoter for *spx* (σP_M_-*spx*) or the *sasA* or *secDF* genes. D) *B*. *subtilis yhdK* null mutant vs. strains additionally mutant for the indicated gene. E) WT *B*. *subtilis* 168 vs. strains lacking the σ^M^-regulated promoter (P_M_) for the indicated gene(s). The percentage change relative to the average colony size is shown above each Box and Whisker plot.
(TIF)

S1 Table Genes compared between reference genomes of *B*. *subtilis* 168 (NCBI accession number NC_000964.3) and PY79 (NC_022898.1). No mutations were found in these genes.
(PDF)

S2 Table Whole genome sequencing of transformants generated by congression. (PDF)

S3 Table Amino acid substitutions in YidC1 variants selected in *yhdL* depletion strain. (PDF)

S4 Table Secreted and membrane-associated proteins in the σ^M^ regulon. (PDF)

S5 Table *Bacillus subtilis* strains used in this study. (PDF)

S6 Table Primers used in this study. (PDF)
