# Supplementary figures and images for: Mutations of the Bacillus subtilis YidC1 (SpoIIIJ) insertase alleviate stress associated with σM-dependent membrane protein overproduction
Source: PLoS Genet. 2019 Oct 18;15(10):e1008263. doi: 10.1371/journal.pgen.1008263 (PMC6827917; doi:10.1371/journal.pgen.1008263)

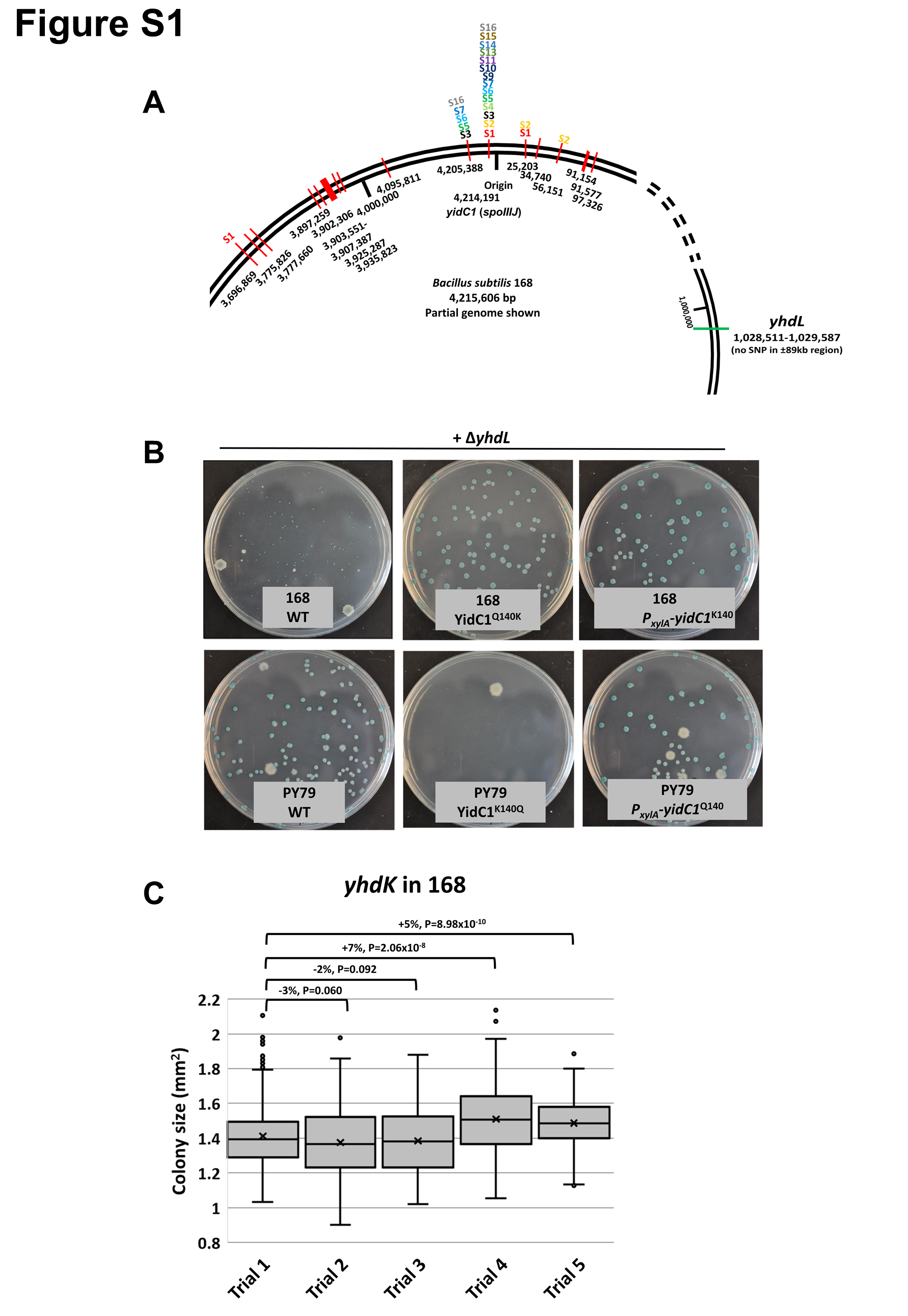

Supplement: S1 Fig — A) Map of SNPs from strain PY79 to 168 and distribution of SNPs contained in each congression suppressors. Genome coordinates were based on the 168 reference genome with NCBI accession number NC_000964.3. B) Transformation plates of yhdL::kan allele transformed into different strain backgrounds, selected on LB plates supplemented with kanamycin, X-gal and 1% xylose. C) Variation of yhdK colony size measurement between trials on different days with different batches of LB plates. P value was calculated using Student’s t test, and percentage changes of average colony size were shown. (TIF) [file pgen.1008263.s001.tif]

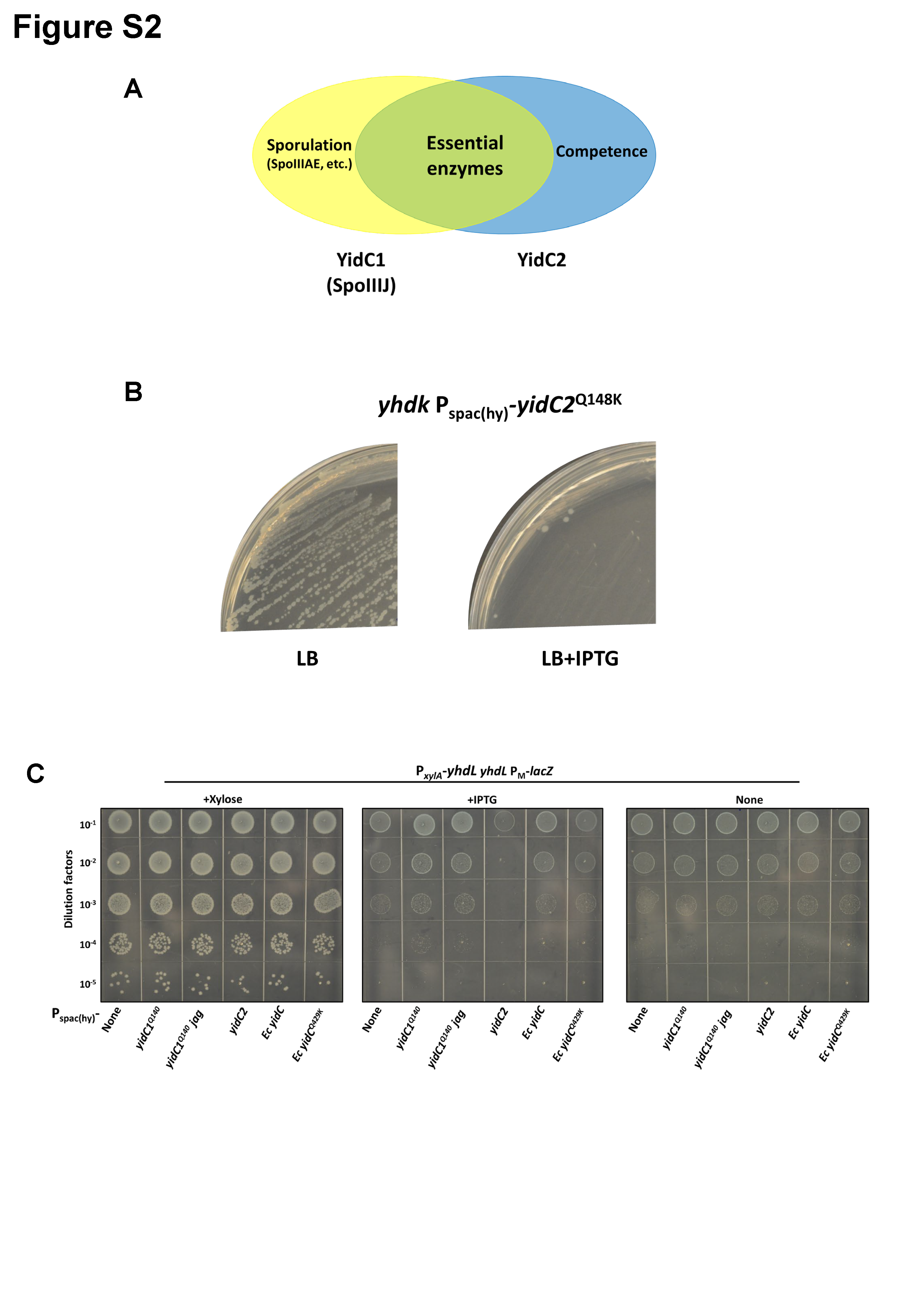

Supplement: S2 Fig — A) Venn diagram of function overlap and distinction of YidC1 and YidC2 of Bacillus subtilis. B) Streaking of yhdK Pspac(hy)-yidC2Q148K on plates of LB or LB supplemented with 1 mM IPTG (final concentration). C) Spot dilution of yhdL depletion strains with Pspac(hy) based overexpression of different YidC homologs, on LB plates supplemented with a final concentration of 1% xylose (+Xylose), 1 mM IPTG (+IPTG) or nothing (None). (TIF) [file pgen.1008263.s002.tif]

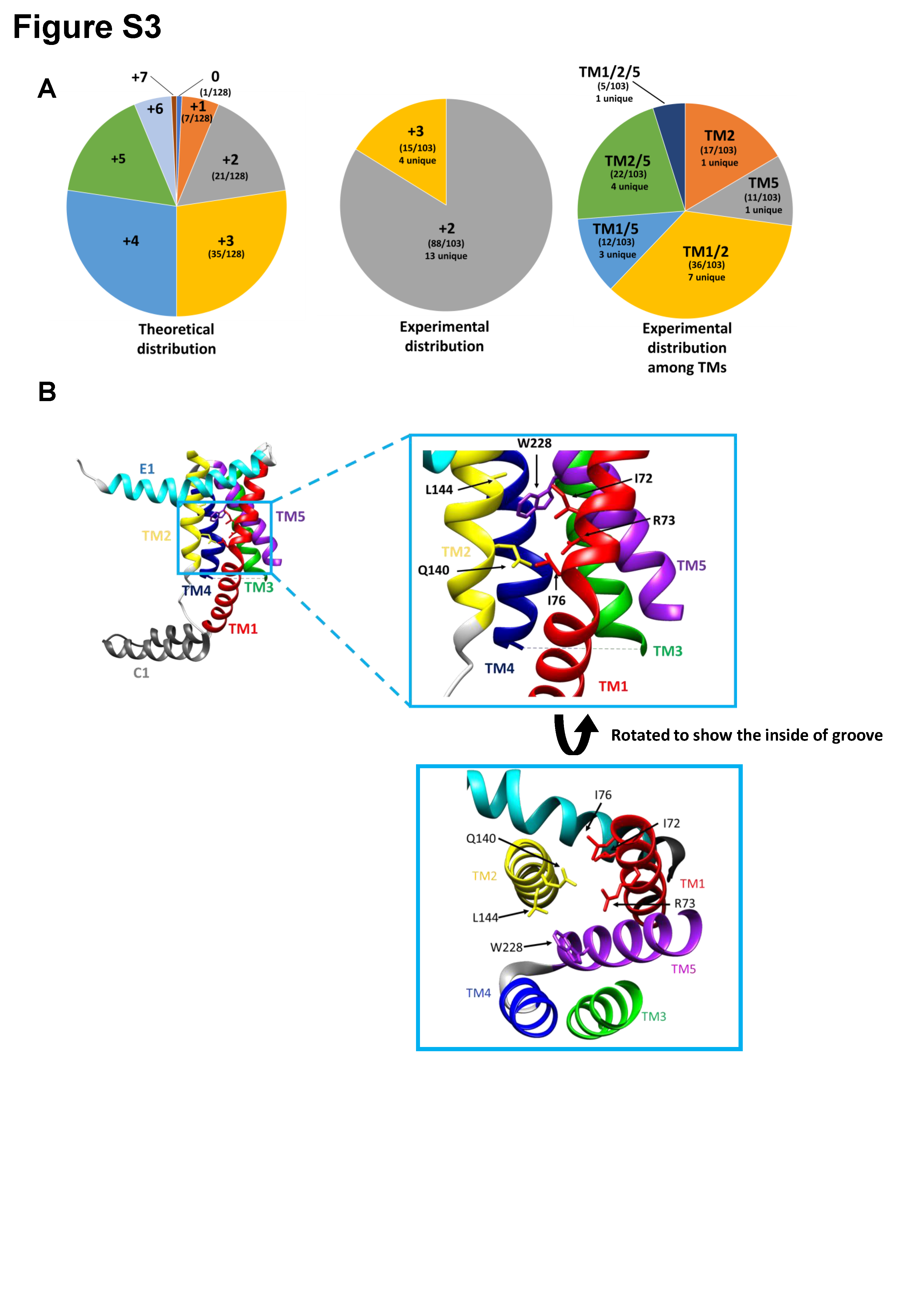

Supplement: S3 Fig — A) Distribution of 128 possible charge variants of YidC1 in the tested library. Theoretically, the majority of YidC1 variants in the input library have a charge of +2 to +5 in the hydrophilic groove, while experimental data from 103 samples suggests that the ones capable of providing high σM tolerance contain a charge of +2 or +3. Among the 103 samples with charge of +2 or +3, the positive charge can be located in one, two or three transmembrane segments, with the exception that no sample contains more than one positive charge in TM1 alone. B) Crystal structure of YidC from Bacillus halodurans (PDB ID 3WO6), showing the seven variable amino acid providing positive charges in the hydrophilic substrate binding chamber of the enzyme. Gly231 is not visible due to the lack of side chain of this residue. TM1-5, transmembrane region 1–5; E1, extracytoplasmic region 1; C1, cytoplasmic region 1.This figure was generated using UCSF Chimera 1.13[49]. (TIF) [file pgen.1008263.s003.tif]

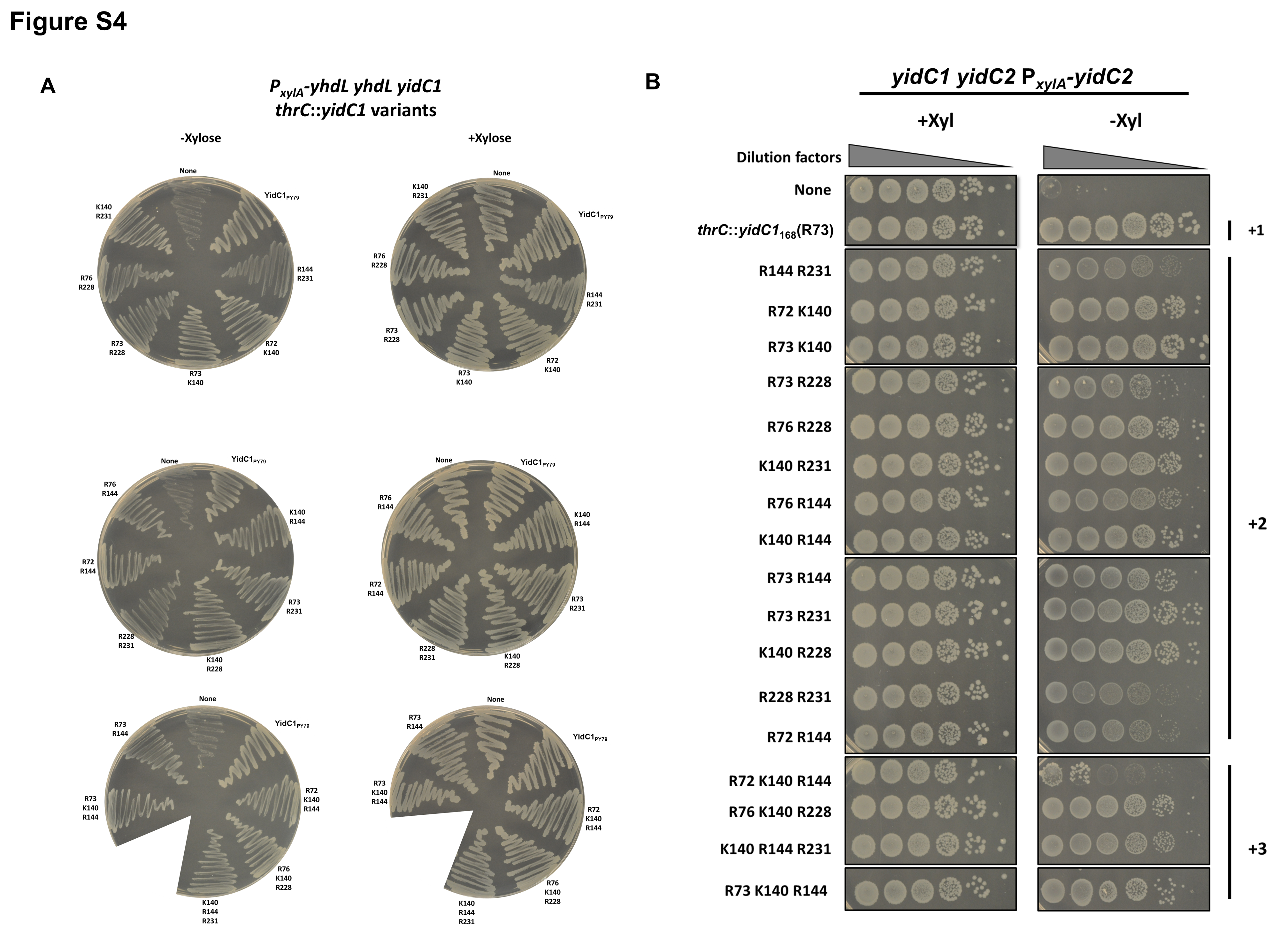

Supplement: S4 Fig — A) yhdL depletion strains with YidC1 variants were streaked on LB plates with or without xylose inducer for yhdL. The positive charge of each variant was labelled next to the streaking, with a negative control “None” meaning no yidC1 variant at thrC locus (weak growth due to the depletion conditions, and cannot be restreaked), and a positive control “YidC1PY79” meaning the native YidC1 mutated into the PY79 Q140K version (HB23719). B) Spot dilution of YidC depletion strains with yidC1 variants at thrC locus. The depletion strain has its native yidC1 and yidC2 deleted, and a xylose inducible copy of PxylA-yidC2. Diluted cultures were spotted on LB without xylose (-Xyl) or with 1% final concentration of xylose (+Xyl). The negative control (None) has no yidC1 at thrC locus, while the positive control has the 168 version of yidC1 that contains a single positive charge at R73. Some YidC1 variants exhibited reduced growth ability, and the variant containing R72 K140 R144 failed to grow, although the emergence of suppressors was noted. (TIF) [file pgen.1008263.s004.tif]

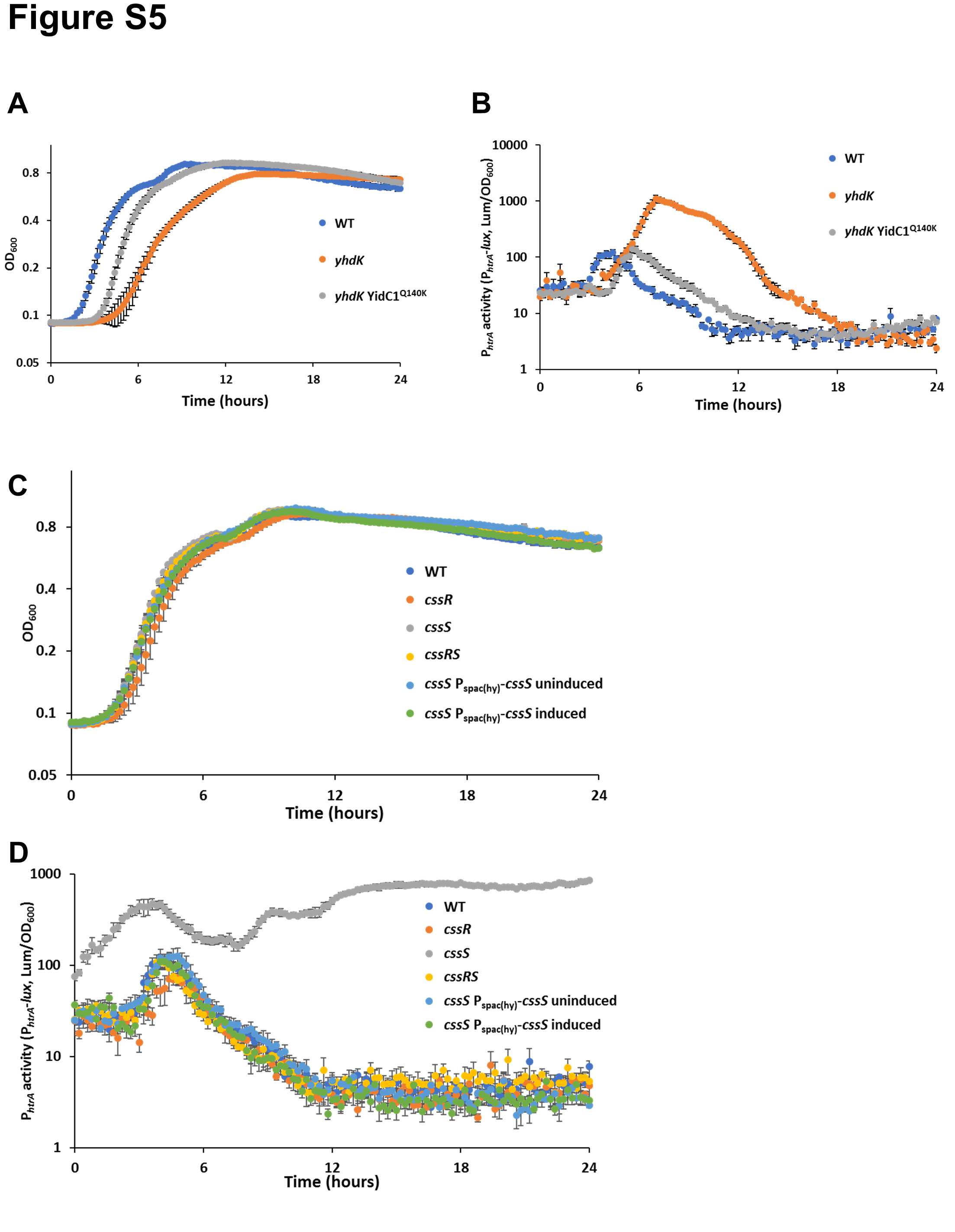

Supplement: S5 Fig — A) Growth curves of WT strain 168, yhdK null, and yhdK with YidC1Q140K single amino acid substitution. B) PhtrA activity of strains in panel A during growth. C) Growth curves of WT strain 168, cssR null, cssS null, cssRS double null, and cssS null with an ectopic IPTG inducible copy under induced or uninduced conditions. D) PhtrA activity of strains in panel C during growth. The OD600 and luminescence were measured every 12 minutes. At least four biological replicates were used for each strain, and the results are shown as mean ± SEM. (TIF) [file pgen.1008263.s005.tif]

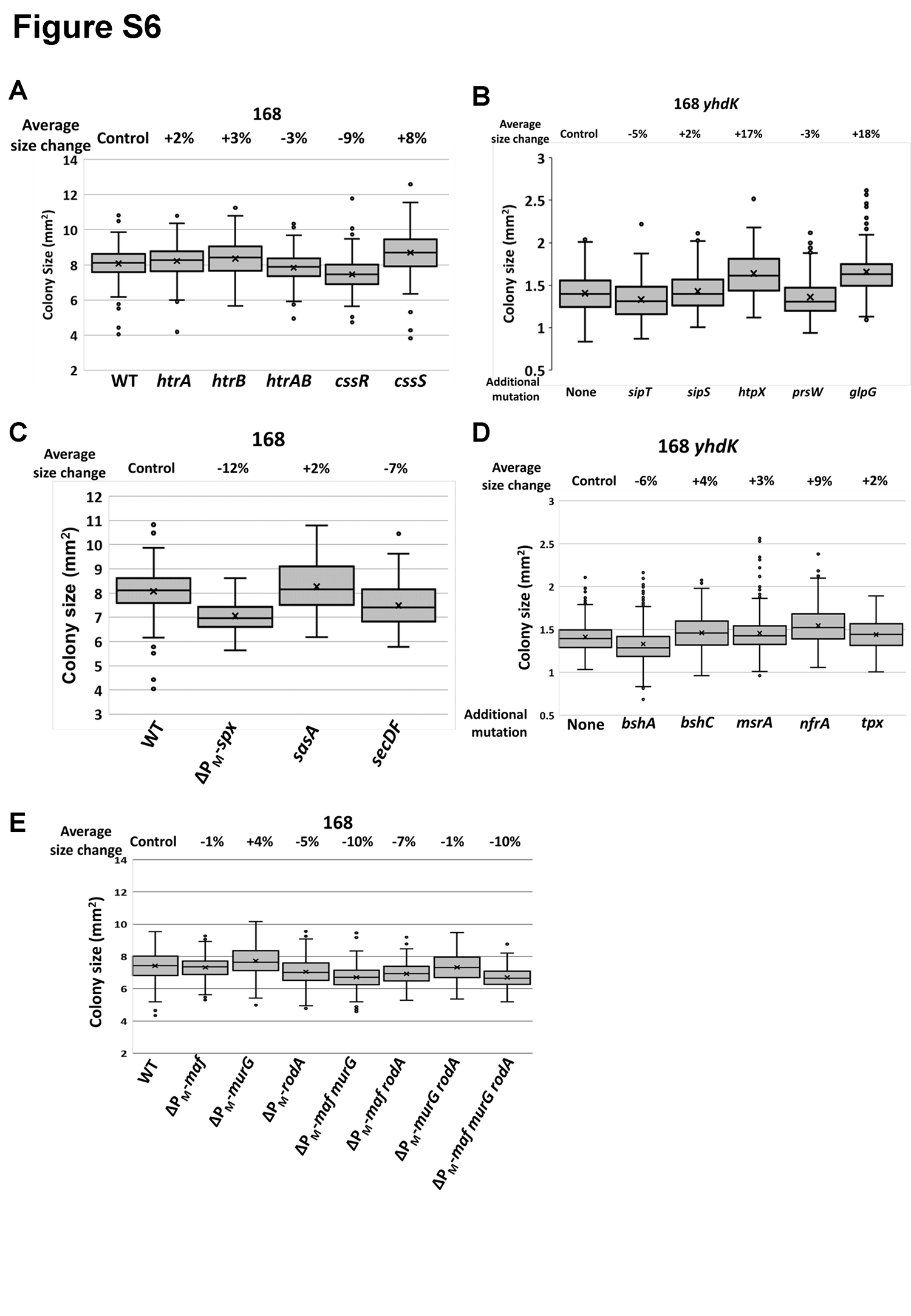

Supplement: S6 Fig — A) WT B. subtilis 168 vs. strains with the indicated mutations. B) B. subtilis yhdK null mutant vs. strains additionally mutant for the indicated gene. C) WT B. subtilis 168 vs. strains lacking either the σM-regulated promoter for spx (σPM-spx) or the sasA or secDF genes. D) B. subtilis yhdK null mutant vs. strains additionally mutant for the indicated gene. E) WT B. subtilis 168 vs. strains lacking the σM-regulated promoter (PM) for the indicated gene(s). The percentage change relative to the average colony size is shown above each Box and Whisker plot. (TIF) [file pgen.1008263.s006.tif]
