# Supplementary material for: Mutations of the Bacillus subtilis YidC1 (SpoIIIJ) insertase alleviate stress associated with σM-dependent membrane protein overproduction
Source: PLoS Genet. 2019 Oct 18;15(10):e1008263. doi: 10.1371/journal.pgen.1008263 (PMC6827917; doi:10.1371/journal.pgen.1008263)
Supplement: S1 Table — No mutations were found in these genes. (PDF) [file pgen.1008263.s007.pdf]

Table S1. Genes compared between reference genomes of *B. subtilis* 168 (NCBI accession number NC_000964.3) and PY79 (NC_022898.1). No SNP was found in these genes.

Accession Number	Gene
**RNAP subunits**	
BSU01430	*rpoA*
BSU01070	*rpoB*
BSU01080	*rpoC*
BSU37160	*rpoE*
BSU14540	*rpoY*
BSU15690	*rpoZ*
**SigM regulon**	
BSU00700	*coaX*
BSU00620	*divIC*
BSU00880	*disA*
BSU38500	*dltA*
BSU38510	*dltB*
BSU38520	*dltC*
BSU38530	*dltD*
BSU38540	*dltE*
BSU00690	*ftsH*
BSU00680	*hprT*
BSU21910	*metA*
BSU28000	*minC*
BSU27990	*minD*
BSU28030	*mreB*
BSU28020	*mreC*
BSU28010	*mreD*
BSU16950	*pbpX*
BSU36380	*rapD*
BSU27650	*secDF*
BSU35650	*tagU*
BSU00670	*tilS*
BSU21920	*ugtP*
BSU00710	*yacC*
BSU00890	*yacL*
BSU00900	*ispD*
BSU02890	*yceC*
BSU02900	*yceD*
BSU02910	*yceE*
BSU02920	*yceF*
BSU02930	*yceG*
BSU02940	*yceH*
BSU03240	*ycgQ*
BSU03250	*ycgR*
BSU04230	*ydaH*
BSU04560	*ddl*
BSU04570	*murF*
BSU06380	*yebC*
BSU07260	*yfnI*
BSU09500	*yhdK*
BSU09510	*yhdL*
BSU09520	*sigM*
BSU11490	*yjbC*
BSU11500	*spx*
BSU14480	*abh*
BSU15230	*murB*
BSU15240	*divIB*
BSU15250	*ylxW*
BSU15260	*ylxX*
BSU15270	*sbp*
BSU18190	*yngC*
BSU22310	*recU*
BSU22320	*ponA*
BSU22980	*ypbG*
BSU23300	*ypuD*
BSU23370	*ypuA*
BSU23830	*yqjL*
BSU27180	*yrhH*
BSU27160	*yrhJ*
BSU27170	*fatR*
BSU28040	*radC*
BSU30500	*ytpB*
BSU30510	*ytpA*
BSU35840	*tagT*
BSU36530	*bcrC*
BSU38120	*rodA*
BSU38480	*ywaC*
BSU38260	*ywbN*
BSU38250	*ywbO*
BSU36540	*ywnJ*
**Spx regulon**	
BSU00730	*cysK*
BSU14710	*ylaA*
BSU14720	*ylaB*
BSU14730	*ylaC*
BSU14740	*ylaD*
BSU21680	*msrB*
BSU21690	*msrA*
BSU27240	*yrhC*
BSU27250	*mccB*
BSU27260	*mccA*
BSU27270	*mtnN*
BSU27280	*yrrT*
BSU28500	*trxA*
BSU29490	*tpx*
BSU31290	*yugT*
BSU31370	*yugJ*
BSU30340	*ytvA*
BSU34790	*trxB*
BSU38110	*nfrA*
BSU29820	*ytpR*
BSU29830	*ytpQ*
BSU29840	*ytpP*
BSU03480	*srfAA*
BSU03490	*srfAB*
BSU03500	*comS*
BSU03510	*srfAC*
BSU03520	*srfAD*
