# Supplementary material for: Mutations of the Bacillus subtilis YidC1 (SpoIIIJ) insertase alleviate stress associated with σM-dependent membrane protein overproduction
Source: PLoS Genet. 2019 Oct 18;15(10):e1008263. doi: 10.1371/journal.pgen.1008263 (PMC6827917; doi:10.1371/journal.pgen.1008263)
Supplement: S2 Table — (PDF) [file pgen.1008263.s008.pdf]

Table S2. Whole genome sequencing of transformants generated by congression

Position	Reference	Allele	Count	Coverage	Frequency	Gene	DNA change	Amino acid change
**Supp-1**								
25203	G	A	72	75	96			
96240	G	A	86	138	62.32			
96260	G	A	65	122	53.28			
486574	T	C	68	69	98.55	*ydaO*	143T>C	Ile48Thr
1297396	G	A	73	73	100	*yjlA*	199C>T	Pro67Ser
2277179	A	C	83	83	100	*yokI*	243T>G	Ile81Met
2277245	GT	AC	36	64	56.25	*yokI*	176_177delACinsGT	Asp59Gly
3391676	A	G	41	41	100	*gerAA*	895A>G	Thr299Ala
3696869	T	C	97	97	100	*pgdS*	630A>G	
4214191	G	T	63	63	100	*yidC1*	418C>A	Gln140Lys
**Supp-2**								
25203	G	A	61	61	100			
56151	G	A	67	67	100	*spoVG*	286G>A	Ala96Thr
455105	T	C	14	25	56	*yczH*	156A>G	
4214191	G	T	50	50	100	*yidC1*	418C>A	Gln140Lys

Position	Reference	Allele	Count	Coverage	Frequency	Gene	DNA change	Amino acid change
**Supp-3**								
4205388	G	A	101	101	100	*yyaC*	489G>A	
4214191	G	T	60	60	100	*yidC1*	418C>A	Gln140Lys
**Supp-4**								
4214191	G	T	49	49	100	*yidC1*	418C>A	Gln140Lys
**Supp-5**								
2600044	TATTGTATAATG	-	42	43	97.67			
4205388	G	A	79	79	100	*yyaC*	489G>A	
4214191	G	T	67	68	98.53	*yidC1*	418C>A	Gln140Lys
**Supp-6**								
4205388	G	A	97	97	100	*yyaC*	489G>A	
4214191	G	T	67	69	97.1	*yidC1*	418C>A	Gln140Lys

Position	Reference	Allele	Count	Coverage	Frequency	Gene	DNA change	Amino acid change
**Supp-7**								
4205388	G	A	72	72	100	*yyaC*	489G>A	
4214191	G	T	47	47	100	*yidC1*	418C>A	Gln140Lys
**Supp-9**								
4214191	G	T	11	11	100	*yidC1*	418C>A	Gln140Lys
**Supp-10**								
4214191	G	T	51	51	100	*yidC1*	418C>A	Gln140Lys
**Supp-11**								
2002473	T	G	85	87	97.7			
4214191	G	T	71	71	100	*yidC1*	418C>A	Gln140Lys
**Supp-12**								
2707867	A	C	59	86	68.6			

Position	Reference	Allele	Count	Coverage	Frequency	Gene	DNA change	Amino acid change
**Supp-13**								
3810012	G	A	128	129	99.22			
4214191	G	T	47	47	100	*yidC1*	418C>A	Gln140Lys
**Supp-14**								
96240	G	A	16	17	94.12			
96260	G	A	13	14	92.86			
775706	-	T	18	32	56.25	*yesZ*	907_908insT	Ala303fs
775707	C	T	18	32	56.25	*yesZ*	909C>T	
775710	-	T	17	33	51.52	*yesZ*	911_912insT	Leu305fs
775721	CC	AA	12	28	42.86	*yesZ*	923_924delCCinsAA	Ser308*
1013666	G	T	15	28	53.57	*nsrR*	186C>A	
1855412	A	T	17	34	50	*pksR*	4523A>T	Asn1508Ile
1855416	A	T	18	36	50	*pksR*	4527A>T	Gln1509His
1855428	T	G	21	40	52.5	*pksR*	4539T>G	Ile1513Met
2129223	G	C	37	37	100	*yodE*	771C>G	
2846430	T	A	11	21	52.38	*nadA*	632A>T	Glu211Val

Position	Reference	Allele	Count	Coverage	Frequency	Gene	DNA change	Amino acid change
**Supp-14 (continued)**								
3510969	AA	GC	14	41	34.15	*yvfH*	190_191delAAinsGC	Lys64Ala
3510977	G	A	21	40	52.5	*yvfH*	198G>A	
3510979	T	A	20	40	50	*yvfH*	200T>A	Met67Lys
4214191	G	T	31	31	100	*yidC1*	418C>A	Gln140Lys
**Supp-15**								
1976137	C	T	134	134	100	*ppsC*	6412G>A	Asp2138Asn
4214191	G	T	49	49	100	*yidC1*	418C>A	Gln140Lys
**Supp-16**								
4205388	G	A	93	94	98.94	*yyaC*	489G>A	
4214191	G	T	59	59	100	*yidC1*	418C>A	Gln140Lys

*Suppressor 8 is not listed here as it contains over 900 SNPs compared with strain 168, and is possibly a contamination of PY79 endospore that survived genomic DNA extraction.

**Suppressor 12 has only a single SNP, and the basis of suppression in this strain is unknown.
