# Supplementary material for: Mutations of the Bacillus subtilis YidC1 (SpoIIIJ) insertase alleviate stress associated with σM-dependent membrane protein overproduction
Source: PLoS Genet. 2019 Oct 18;15(10):e1008263. doi: 10.1371/journal.pgen.1008263 (PMC6827917; doi:10.1371/journal.pgen.1008263)
Supplement: S3 Table — (PDF) [file pgen.1008263.s009.pdf]

Table S3. Amino acid substitutions in YidC1 variants selected in *yhdL* depletion strain

Suppressor	Total Charge	Ile72Arg	Gln73Arg	Ile76Arg	Gln140Lys	Leu144Arg	Trp228Arg	Gly231Arg
1	3				1	1		1
2	2						1	1
3	2				1	1		
4	2					1		1
5	2						1	1
6	3	1			1	1		
7	3	1			1	1		
8	2	1			1			
9	2		1				1	
10	2	1			1			
11	2		1		1			
12	2				1		1	
13	2	1			1			
14	2			1		1		
15	2			1			1	
16	2				1		1	
17	2				1		1	
18	2						1	1
19	3				1	1		1
20	2		1				1	
21	3			1	1		1	
22	2						1	1
23	2		1				1	
24	2			1			1	
25	2						1	1
26	2				1		1	
27	2	1			1			
28	3			1	1		1	
29	2		1		1			
30	2		1				1	
31	2	1			1			
32	2				1			1
33	3	1			1	1		
34	2						1	1
35	2			1			1	
36	2			1		1		
37	3				1	1		1
38	2				1	1		
39	2			1		1		
40	2		1			1		
41	2				1			1

Suppressor	Total Charge	Ile72Arg	Gln73Arg	Ile76Arg	Gln140Lys	Leu144Arg	Trp228Arg	Gly231Arg
42	2		1					1
43	2			1		1		
44	2				1	1		
45	2			1		1		
46	2	1				1		
47	2	1			1			
48	2						1	1
49	2		1		1			
50	2		1				1	
51	2				1		1	
52	2	1			1			
53	2				1		1	
54	2				1		1	
55	2				1		1	
56	2						1	1
57	2				1		1	
58	2				1			1
59	3			1	1		1	
60	2	1				1		
61	2				1		1	
62	2						1	1
63	2				1	1		
64	2			1		1		
65	2		1			1		
66	3			1	1		1	
67	2				1	1		
68	2		1		1			
69	2						1	1
70	2				1	1		
71	2				1	1		
72	2						1	1
73	2				1		1	
74	2		1			1		
75	2				1		1	
76	2				1	1		
77	2				1		1	
78	2				1	1		
79	2				1		1	
80	2				1	1		
81	2			1			1	
82	2	1			1			
83	2				1	1		
84	2	1			1			

Suppressor	Total Charge	Ile72Arg	Gln73Arg	Ile76Arg	Gln140Lys	Leu144Arg	Trp228Arg	Gly231Arg
85	2		1				1	
86	2				1	1		
87	2		1				1	
88	2				1	1		
89	2				1	1		
90	2				1		1	
91	2				1	1		
92	2	1			1			
93	2		1		1			
94	2			1		1		
95	3		1		1	1		
96	3			1	1		1	
97	2				1	1		
98	3		1		1	1		
99	2				1	1		
100	2		1		1			
101	2			1		1		
102	2	1				1		
103	3		1		1	1		
