# Supplementary material for: Mutations of the Bacillus subtilis YidC1 (SpoIIIJ) insertase alleviate stress associated with σM-dependent membrane protein overproduction
Source: PLoS Genet. 2019 Oct 18;15(10):e1008263. doi: 10.1371/journal.pgen.1008263 (PMC6827917; doi:10.1371/journal.pgen.1008263)
Supplement: S4 Table — (PDF) [file pgen.1008263.s010.pdf]

Table S4. Secreted and membrane-associated proteins in the σ^M regulon

Locus	Gene	Protein Localization	Number of predicted transmembrane segments
BSU00570	*yabM*	cytoplasmic membrane	14
BSU00600	*yabP*	forespore outer membrane	Unknown
BSU00610	*yabQ*	forespore outer membrane	6
BSU00620	*divIC*	peripheral membrane protein	Peripheral
BSU00690	*ftsH*	cytoplasmic membrane	2
BSU02900	*yceD*	peripheral membrane protein	Peripheral
BSU02920	*yceF*	cytoplasmic membrane	6
BSU03240	*ycgQ*	cytoplasmic membrane	4
BSU03250	*ycgR*	cytoplasmic membrane	8
BSU04230	*amj*	cytoplasmic membrane	7
BSU06380	*yebC*	cytoplasmic membrane	5
BSU09500	*yhdK*	cytoplasmic membrane	3
BSU09510	*yhdL*	cytoplasmic membrane	1
BSU15220	*murG*	peripheral membrane protein	Peripheral
BSU15240	*divIB*	cytoplasmic membrane	1
BSU15250	*ylxW*	Integral membrane protein	Unknown
BSU15260	*ylxX*	cytoplasmic membrane	1
BSU15270	*sbp*	cytoplasmic membrane	3
BSU15280	*ftsA*	cytoplasmic membrane and cytoplasm	Peripheral
BSU15290	*ftsZ*	cytoplasmic membrane and cytoplasm	Peripheral
BSU18190	*yngC*	cytoplasmic membrane	4
BSU21920	*ugtP*	peripheral membrane protein	Peripheral
BSU22320	*ponA*	cytoplasmic membrane	1
BSU27160	*yrhJ*	cytoplasmic membrane	Unknown
BSU27650	*secDF*	cytoplasmic membrane	12
BSU27990	*minD*	peripheral membrane protein	Peripheral
BSU28000	*minC*	peripheral membrane protein	Peripheral
BSU28010	*mreD*	cytoplasmic membrane	5
BSU28020	*mreC*	cytoplasmic membrane	1
BSU28030	*mreB*	peripheral membrane protein	Peripheral
BSU35650	*tagU*	cytoplasmic membrane	1
BSU35840	*tagT*	cytoplasmic membrane	1
BSU36530	*bcrC*	cytoplasmic membrane	4
BSU38120	*rodA*	cytoplasmic membrane	11
BSU38260	*efeB*	Secreted protein	Secreted
BSU38270	*efeO*	cytoplasmic membrane	Peripheral
BSU38280	*efeU*	cytoplasmic membrane	6
BSU38510	*dltB*	cytoplasmic membrane	10
