# Supplementary material for: Mutations of the Bacillus subtilis YidC1 (SpoIIIJ) insertase alleviate stress associated with σM-dependent membrane protein overproduction
Source: PLoS Genet. 2019 Oct 18;15(10):e1008263. doi: 10.1371/journal.pgen.1008263 (PMC6827917; doi:10.1371/journal.pgen.1008263)
Supplement: S5 Table — (PDF) [file pgen.1008263.s011.pdf]

Table S5. *Bacillus subtilis* strains used in this study

Number	Genotype	Note or Reference
HB16780	ΔP_M-*murG*	Mutated *sigM* promoter inside *murG* gene using vector pMutin4
HB16812	ΔP_M-*murG* ΔP_M-*maf*	
HB17934	ΔP_M-*maf*	Mutated *sigM* promoter inside *maf* gene using vector pMutin4
HB18905	*spx*::P_{spx}(P_{M1}*)-*spx* (*kan*)	From (1)
HB20830	*yhdK*::*erm*	All gene::*erm* and gene::*kan* strains were constructed as using genomic DNA of BKE or BKK strains into recipient strains (2)
HB20922	*spx*::P_{spx}(P_{M1}*)-*spx* (*kan*) *yhdK*::*erm*	
HB21099	ΔP_M-*rodA*	P_M promoter of *rodA* removed using CRISPR
HB21117	ΔP_M-*rodA* ΔP_M-*murG*	ΔP_M-*murG* transformed with CRISPR plasmid to remove P_M of *rodA*
HB21118	ΔP_M-*rodA* ΔP_M-*maf*	ΔP_M-*maf* transformed with CRISPR plasmid to remove P_M of *rodA*
HB21266	ΔP_M-*maf* ΔP_M-*murG* ΔP_M-*rodA*	P_M promoter of *rodA* removed using CRISPR from ΔP_M-*maf* ΔP_M-*murG* background
HB25433	ΔP_M-*maf yhdK*::*erm*	
HB25434	ΔP_M-*murG yhdK*::*erm*	
HB25435	ΔP_M-*rodA yhdK*::*erm*	
HB25436	ΔP_M-*murG* ΔP_M-*maf yhdK*::*erm*	
HB25437	ΔP_M-*rodA* ΔP_M-*maf yhdK*::*erm*	
HB25438	ΔP_M-*rodA* ΔP_M-*murG yhdK*::*erm*	
HB25439	ΔP_M-*maf* ΔP_M-*murG* ΔP_M-*rodA yhdK*::*erm*	
HB21248	PY79 P_M-*lacZ yhdL*::*kan*	PY79 background, *yhdL* is not essential in PY79 background
HB21258	168 P_M-*lacZ yhdL*::*kan* congression from PY79-1	
HB21259	168 P_M-*lacZ yhdL*::*kan* congression from PY79-2	
HB21260	168 P_M-*lacZ yhdL*::*kan* congression from PY79-3	
HB21261	168 P_M-*lacZ yhdL*::*kan* congression from PY79-4	
HB21262	168 P_M-*lacZ yhdL*::*kan* congression from PY79-5	
HB21263	168 P_M-*lacZ yhdL*::*kan* congression from PY79-6	
HB21264	168 P_M-*lacZ yhdL*::*kan* congression from PY79-7	
HB21265	168 P_M-*lacZ yhdL*::*kan* congression from PY79-8	Not included in Figure S1, as this strain contains too many SNPs from PY79, possible endospore contamination from PY79 genomic DNA prep
HB21250	168 P_M-*lacZ yhdL*::*kan* congression from PY79-9	
HB21251	168 P_M-*lacZ yhdL*::*kan* congression from PY79-10	

HB21252	168 P_M-*lacZ yhdL*::*kan* congression from PY79-11	
HB21253	168 P_M-*lacZ yhdL*::*kan* congression from PY79-12	
HB21254	168 P_M-*lacZ yhdL*::*kan* congression from PY79-13	
HB21255	168 P_M-*lacZ yhdL*::*kan* congression from PY79-14	
HB21256	168 P_M-*lacZ yhdL*::*kan* congression from PY79-15	
HB21257	168 P_M-*lacZ yhdL*::*kan* congression from PY79-16	
HB22728	*yidC1*	
HB22789	P_M-*lacZ* *yidC1*^{Q140K} *yhdL*::*kan*	
HB22848	*yidC1*^{Q140K} *yhdK*::*erm*	
HB22849	PY79 P_M-*lacZ yidC1*^{K140Q} *yhdK*::*erm*	
HB22850	PY79 P_M-*lacZ yidC1 yhdK*::*erm*	
HB22883	PY79 *yhdK*::*erm*	
HB22925	PY79 P_M-*lacZ yidC1*^{K140Q}	
HB22926	PY79 P_M-*lacZ yidC1*	
HB22966	*yidC1*^{Q140K}	*yidC1* was mutated at its native locus using CRISPR
HB23553	*amyE*::$P_{spac(hy)}$-*yidC1*	*amyE*::$P_{spac(hy)}$ constructs were made using plasmid pPL82
HB23556	*amyE*::$P_{spac(hy)}$-*yidC2*	
HB23558	*amyE*::$P_{spac(hy)}$-*E. coli-yidC*	
HB23595	*amyE*::$P_{spac(hy)}$-*E. coli-yidC*^{Q429K}	
HB25405	*amyE*::$P_{spac(hy)}$-*yidC1 yhdK*::*erm*	
HB25406	*amyE*::$P_{spac(hy)}$-*yidC2 yhdK*::*erm*	
HB25407	*amyE*::$P_{spac(hy)}$-*E.coli-yidC yhdK*::*erm*	
HB25408	*amyE*::$P_{spac(hy)}$-*E.coli-yidC*^{Q429K} *yhdK*::*erm*	
HB23605	*thrC*::P_M-*spoVG-lacZ*-spec *ganA*::P_{xylA}-*yhdL amyE::yidC1 yhdL*::*kan*	*ganA*::P_{xylA}-*yhdL* was constructed using pAX01, *thrC*::P_M-*spoVG-lacZ* reporter was constructed using pDG1663, with the *Erm*^{R} cassette replaced by a Spec^R cassette using LFH PCR
HB23606	*thrC*::P_M-*spoVG-lacZ*-spec *ganA*::P_{xylA}-*yhdL amyE::yidC1-jag yhdL*::*kan*	
HB23607	*thrC*::P_M-*spoVG-lacZ*-spec *ganA*::P_{xylA}-*yhdL amyE::yidC2 yhdL*::*kan*	
HB23608	*thrC*::P_M-*spoVG-lacZ*-spec *ganA*::P_{xylA}-*yhdL amyE::E. coli-yidC yhdL*::*kan*	
HB23609	*thrC*::P_M-*spoVG-lacZ*-spec *ganA*::P_{xylA}-*yhdL amyE:: E. coli-yidC*^{Q429K} *yhdL*::*kan*	
HB23610	*yidC1*^{R73A} *ganA*::P_{xylA}-*yidC2 yidC2*::*kan*	*ganA*::P_{xylA}-*yidC2* was constructed using pAX01
HB23611	*yidC1*^{R73AQ140K} *ganA*::P_{xylA}-*yidC2 yidC2*::*kan*	
HB23698	*yidC1*^{R73AQ140K} *ganA*::P_{xylA}-*yhdL-cat yhdL*::*kan*	*ganA*::P_{xylA}-*yhdL*-cat was constructed using pAX01, and the original *Erm*^{R} cassette in pAX01 was replaced by a CM^R cassette using LFH PCR

HB23719	*yidC1*^{Q140K} *ganA*::P_{xylA}-*yhdL*-cat *yhdL*::*Kan*	
HB23902	*amyE*::$P_{spac(hy)}$-*yidC2*^{Q148K}	
HB25409	*amyE*::$P_{spac(hy)}$- *yidC2*^{Q148K} *yhdK*::*erm*	
HB23917	*yidC2*'-*lacZ*	Genomic DNA of SCB751 (3) transformed into 168
HB23918	*yidC2*'-*lacZ* *yidC1*	Genomic DNA of SCB751 (3) transformed into HB22728
HB23935	*yidC2*'-*lacZ* *yidC1* *thrC*::*yidC1*-spec	
HB23953	*ganA*::P_{xylA}-*yhdL* P_M-*lacZ* *yhdL*::*kan*	
HB23955	*yidC2* *ganA*::P_{xylA}-*yidC2* *yidC1*::*kan*	
HB23965	*thrC*::*yidC1*-spec	*thrC*::*yidC1*-spec WT allele was constructed using LFH PCR
HB23966	P_M-*lacZ* *thrC*::*yidC1*-spec	
HB23967	*yidC1* P_M-*lacZ* *thrC*::*yidC1*-spec	
HB23968	*yidC2* P_{xylA}-*yidC2* *yidC1*::*kan* *thrC*::*yidC1*-spec	
HB23969	*thrC*::*yidC1*^{Q140K}-spec ΔP_M-*maf*	
HB23970	*thrC*::*yidC1*^{Q140K}-spec ΔP_M-*murG*	
HB23971	*thrC*::*yidC1*^{Q140K}-spec ΔP_M-*rodA*	
HB23972	*thrC*::*yidC1*^{Q140K}-spec ΔP_M-*maf* ΔP_M-*murG*	
HB23973	*thrC*::*yidC1*^{Q140K}-spec ΔP_M-*maf* ΔP_M-*rodA*	
HB23974	*thrC*::*yidC1*^{Q140K}-spec ΔP_M-*murG* ΔP_M-*rodA*	
HB23975	*thrC*::*yidC1*^{Q140K}-spec ΔP_M-*maf* ΔP_M-*murG* ΔP_M-*rodA*	
HB25440	*thrC*::*yidC1*^{Q140K}-spec ΔP_M-*maf* *yhdK*::*erm*	
HB25441	*thrC*::*yidC1*^{Q140K}-spec ΔP_M-*murG* *yhdK*::*erm*	
HB25442	*thrC*::*yidC1*^{Q140K}-spec ΔP_M-*rodA* *yhdK*::*erm*	
HB25443	*thrC*::*yidC1*^{Q140K}-spec ΔP_M-*maf* ΔP_M-*murG* *yhdK*::*erm*	
HB25444	*thrC*::*yidC1*^{Q140K}-spec ΔP_M-*maf* ΔP_M-*rodA* *yhdK*::*erm*	
HB25445	*thrC*::*yidC1*^{Q140K}-spec ΔP_M-*murG* ΔP_M-*rodA* *yhdK*::*erm*	
HB25446	*t thrC*::*yidC1*^{Q140K}-spec ΔP_M-*maf* ΔP_M-*murG* ΔP_M-*rodA* *yhdK*::*erm*	
HB23976	*thrC*::*yidC1*-spec WT (R73)	
HB23977	P_M-*lacZ* *thrC*::*yidC1*-spec WT (R73)	
HB23978	*yidC1* P_M-*lacZ* *thrC*::*yidC1*-spec WT (R73)	
HB25354	*yidC1* P_M-*lacZ* *thrC*::*yidC1*-spec sup (R144R231)	*thrC*::*yidC1*-spec allele variants were constructed using degenerative primers and LFH PCR
HB25355	*yidC1* P_M-*lacZ* *thrC*::*yidC1*-spec sup (R72K140)	
HB25356	*yidC1* P_M-*lacZ* *thrC*::*yidC1*-spec sup (R73R228)	

HB25357	*yidC1* P_M-*lacZ thrC*::*yidC1*-spec sup (K140R231)
HB25358	*yidC1* P_M-*lacZ thrC*::*yidC1*-spec sup (R76R144)
HB25359	*yidC1* P_M-*lacZ thrC*::*yidC1*-spec sup (R73R144)
HB25360	*yidC1* P_M-*lacZ thrC*::*yidC1*-spec sup (R73R231)
HB25361	*yidC1* P_M-*lacZ thrC*::*yidC1*-spec sup (K140R228)
HB25362	*yidC1* P_M-*lacZ thrC*::*yidC1*-spec sup (R228R231)
HB25363	*yidC1* P_M-*lacZ thrC*::*yidC1*-spec sup (R72R144)
HB25364	*yidC1* P_M-*lacZ thrC*::*yidC1*-spec sup (K140R144R231)
HB25366	*yidC1* P_M-*lacZ thrC*::*yidC1*-spec sup (R73K140R144)
HB25376	*yhdK*::*erm yidC1* P_M-*lacZ thrC*::*yidC1*-spec sup (R144R231)
HB25377	*yhdK*::*erm yidC1* P_M-*lacZ thrC*::*yidC1*-spec sup (R72K140)
HB25378	*yhdK*::*erm yidC1* P_M-*lacZ thrC*::*yidC1*-spec sup (R73R228)
HB25379	*yhdK*::*erm yidC1* P_M-*lacZ thrC*::*yidC1*-spec sup (K140R231)
HB25380	*yhdK*::*erm yidC1* P_M-*lacZ thrC*::*yidC1*-spec sup (R76R144)
HB25381	*yhdK*::*erm yidC1* P_M-*lacZ thrC*::*yidC1*-spec sup (R73R144)
HB25382	*yhdK*::*erm yidC1* P_M-*lacZ thrC*::*yidC1*-spec sup (R73R231)
HB25383	*yhdK*::*erm yidC1* P_M-*lacZ thrC*::*yidC1*-spec sup (K140R228)
HB25384	*yhdK*::*erm yidC1* P_M-*lacZ thrC*::*yidC1*-spec sup (R228R231)
HB25385	*yhdK*::*erm yidC1* P_M-*lacZ thrC*::*yidC1*-spec sup (R72R144)
HB25386	*yhdK*::*erm yidC1* P_M-*lacZ thrC*::*yidC1*-spec sup (K140R144R231)
HB25387	*yhdK*::*erm yidC1* P_M-*lacZ thrC*::*yidC1*-spec sup (R73K140R144)
HB23987	*yidC1* P_M-*lacZ thrC*::*yidC1*-spec sup (R73K140)
HB23988	*yidC1* P_M-*lacZ thrC*::*yidC1*-spec sup (R76R228)
HB23989	*yidC1* P_M-*lacZ thrC*::*yidC1*-spec sup (K140R144)
HB23990	*yidC1* P_M-*lacZ thrC*::*yidC1*-spec sup (R72K140R144)
HB23991	*yidC1* P_M-*lacZ thrC*::*yidC1*-spec sup (R76K140R228)
HB25388	*yhdK*::*erm yidC1* P_M-*lacZ thrC*::*yidC1*-spec sup (R73K140)
HB25389	*yhdK*::*erm yidC1* P_M-*lacZ thrC*::*yidC1*-spec sup (R76R228)
HB25390	*yhdK*::*erm yidC1* P_M-*lacZ thrC*::*yidC1*-spec sup (K140R144)
HB25391	*yhdK*::*erm yidC1* P_M-*lacZ thrC*::*yidC1*-spec sup (R72K140R144)
HB25392	*yhdK*::*erm yidC1* P_M-*lacZ thrC*::*yidC1*-spec sup (R76K140R228)
HB25404	*yhdK*::*erm yidC1* P_M-*lacZ thrC*::*yidC1*-spec WT (R73)

HB25107	P_M-*lacZ ganA*::P_{xylA}-*yhdL yhdL*::*kan yidC1 thrC*::*yidC1* sup (R144R231)
HB25108	P_M-*lacZ ganA*::P_{xylA}-*yhdL yhdL*::*kan yidC1 thrC*::*yidC1* sup (R72K140)
HB25109	P_M-*lacZ ganA*::P_{xylA}-*yhdL yhdL*::*kan yidC1 thrC*::*yidC1* sup (R73K140)
HB25110	P_M-*lacZ ganA*::P_{xylA}-*yhdL yhdL*::*kan yidC1 thrC*::*yidC1* sup (R73R228)
HB25111	P_M-*lacZ ganA*::P_{xylA}-*yhdL yhdL*::*kan yidC1 thrC*::*yidC1* sup (R76R228)
HB25112	P_M-*lacZ ganA*::P_{xylA}-*yhdL yhdL*::*kan yidC1 thrC*::*yidC1* sup (K140R231)
HB25113	P_M-*lacZ ganA*::P_{xylA}-*yhdL yhdL*::*kan yidC1 thrC*::*yidC1* sup (R76R144)
HB25114	P_M-*lacZ ganA*::P_{xylA}-*yhdL yhdL*::*kan yidC1 thrC*::*yidC1* sup (K140R144)
HB25115	P_M-*lacZ ganA*::P_{xylA}-*yhdL yhdL*::*kan yidC1 thrC*::*yidC1* sup (R73R144)
HB25116	P_M-*lacZ ganA*::P_{xylA}-*yhdL yhdL*::*kan yidC1 thrC*::*yidC1* sup (R73R231)
HB25117	P_M-*lacZ ganA*::P_{xylA}-*yhdL yhdL*::*kan yidC1 thrC*::*yidC1* sup (K140R228)
HB25118	P_M-*lacZ ganA*::P_{xylA}-*yhdL yhdL*::*kan yidC1 thrC*::*yidC1* sup (R228R231)
HB25119	P_M-*lacZ ganA*::P_{xylA}-*yhdL yhdL*::*kan yidC1 thrC*::*yidC1* sup (R72R144)
HB25120	P_M-*lacZ ganA*::P_{xylA}-*yhdL yhdL*::*kan yidC1 thrC*::*yidC1* sup (R72K140R144)
HB25121	P_M-*lacZ ganA*::P_{xylA}-*yhdL yhdL*::*kan yidC1 thrC*::*yidC1* sup (R76K140R228)
HB25122	P_M-*lacZ ganA*::P_{xylA}-*yhdL yhdL*::*kan yidC1 thrC*::*yidC1* sup (K140R144R231)
HB25123	P_M-*lacZ ganA*::P_{xylA}-*yhdL yhdL*::*kan yidC1 thrC*::*yidC1* sup (R73K140R228)
HB25124	P_M-*lacZ ganA*::P_{xylA}-*yhdL yhdL*::*kan yidC1 thrC*::*yidC1* sup (R73K140R144)
HB25227	*yidC2'-lacZ yidC1 thrC*::*yidC1* sup (R144R231)
HB25228	*yidC2'-lacZ yidC1 thrC*::*yidC1* sup (R72K140)
HB25229	*yidC2'-lacZ yidC1 thrC*::*yidC1* sup (R73K140)
HB25230	*yidC2'-lacZ yidC1 thrC*::*yidC1* sup (R73R228)
HB25231	*yidC2'-lacZ yidC1 thrC*::*yidC1* sup (R76R228)
HB25232	*yidC2'-lacZ yidC1 thrC*::*yidC1* sup (K140R231)
HB25233	*yidC2'-lacZ yidC1 thrC*::*yidC1* sup (R76R144)
HB25234	*yidC2'-lacZ yidC1 thrC*::*yidC1* sup (K140R144)
HB25235	*yidC2'-lacZ yidC1 thrC*::*yidC1* sup (R73R144)
HB25236	*yidC2'-lacZ yidC1 thrC*::*yidC1* sup (R73R231)
HB25237	*yidC2'-lacZ yidC1 thrC*::*yidC1* sup (K140R228)
HB25238	*yidC2'-lacZ yidC1 thrC*::*yidC1* sup (R228R231)
HB25239	*yidC2'-lacZ yidC1 thrC*::*yidC1* sup (R72R144)
HB25240	*yidC2'-lacZ yidC1 thrC*::*yidC1* sup (R72K140R144)

HB25241	*yidC2'-lacZ yidC1 thrC*::*yidC1* sup (R76K140R228)	
HB25242	*yidC2'-lacZ yidC1 thrC*::*yidC1* sup (K140R144R231)	
HB25243	*yidC2'-lacZ yidC1 thrC*::*yidC1* sup (R73K140R228)	
HB25244	*yidC2'-lacZ yidC1 thrC*::*yidC1* sup (R73K140R144)	
HB25287	*yidC2 ganA*::P_{xylA}-*yidC2 yidC1*::*kan thrC*::*yidC1* sup (R144R231)	
HB25288	*yidC2 ganA*::P_{xylA}-*yidC2 yidC1*::*kan thrC*::*yidC1* sup (R72K140)	
HB25289	*yidC2 ganA*::P_{xylA}-*yidC2 yidC1*::*kan thrC*::*yidC1* sup (R73K140)	
HB25290	*yidC2 ganA*::P_{xylA}-*yidC2 yidC1*::*kan thrC*::*yidC1* sup (R73R228)	
HB25291	*yidC2 ganA*::P_{xylA}-*yidC2 yidC1*::*kan thrC*::*yidC1* sup (R76R228)	
HB25292	*yidC2 ganA*::P_{xylA}-*yidC2 yidC1*::*kan thrC*::*yidC1* sup (K140R231)	
HB25293	*yidC2 ganA*::P_{xylA}-*yidC2 yidC1*::*kan thrC*::*yidC1* sup (R76R144)	
HB25294	*yidC2 ganA*::P_{xylA}-*yidC2 yidC1*::*kan thrC*::*yidC1* sup (K140R144)	
HB25295	*yidC2 ganA*::P_{xylA}-*yidC2 yidC1*::*kan thrC*::*yidC1* sup (R73R144)	
HB25296	*yidC2 ganA*::P_{xylA}-*yidC2 yidC1*::*kan thrC*::*yidC1* sup (R73R231)	
HB25297	*yidC2 ganA*::P_{xylA}-*yidC2 yidC1*::*kan thrC*::*yidC1* sup (K140R228)	
HB25298	*yidC2 ganA*::P_{xylA}-*yidC2 yidC1*::*kan thrC*::*yidC1* sup (R228R231)	
HB25299	*yidC2 ganA*::P_{xylA}-*yidC2 yidC1*::*kan thrC*::*yidC1* sup (R72R144)	
HB25300	*yidC2 ganA*::P_{xylA}-*yidC2 yidC1*::*kan thrC*::*yidC1* sup (R72K140R144)	
HB25301	*yidC2 ganA*::P_{xylA}-*yidC2 yidC1*::*kan thrC*::*yidC1* sup (R76K140R228)	
HB25302	*yidC2 ganA*::P_{xylA}-*yidC2 yidC1*::*kan thrC*::*yidC1* sup (K140R144R231)	
HB25303	*yidC2 ganA*::P_{xylA}-*yidC2 yidC1*::*kan thrC*::*yidC1* sup (R73K140R228)	
HB25304	*yidC2 ganA*::P_{xylA}-*yidC2 yidC1*::*kan thrC*::*yidC1* sup (R73K140R144)	
HB23636	*htrA*::*kan*	
HB23637	*htrB*::*kan*	
HB23638	*glpG*::*kan*	
HB23639	*sipS*::*kan*	
HB23640	*sipT*::*kan*	
HB23641	*htpX*::*kan*	
HB23648	*htrAB*	
HB23650	*sacA*::P_{htrA}-*lux-cat*	Chloramphenicol^{R} provided by plasmid pBs3Clux
HB25678	*sacA*::P_{htrA}-*lux-erm*	Cam^{R} cassette replaced by Erm^{R} cassette using LFH PCR, Primers 6878-6881
HB23651	*cssR*	

HB23653	*cssS*	
HB23657	*sacA*::P$_{htrA}$-*lux yidC1*^{Q140K}	
HB23660	*amyE*::P$_{spac(hy)}$-none	pPL82 empty vector
HB23663	*amyE*::P$_{spac(hy)}$-*htrA*	constructed using pPL82
HB23664	*amyE*::P$_{spac(hy)}$-*htrB*	constructed using pPL82
HB25679	*sacA*::P$_{htrA}$-*lux-erm cssR*	
HB25680	*sacA*::P$_{htrA}$-*lux-erm cssS*	
HB25681	*sacA*::P$_{htrA}$-*lux-erm cssRS*	
HB25682	*amyE*::P$_{spac(hy)}$-*cssS cssS sacA*::P$_{htrA}$-*lux-erm*	
HB23682	*prsW*::*kan*	
HB23690	*cssS htrB*	
HB23910	*secDF*	
HB23926	*sasA*	
HB23806	*bshC*	
HB23807	*msrA*	
HB23808	*nfrA*	
HB23809	*tpx*	
HB23811	*bshA*	
HB25410	*htrA*::*kan yhdK*::*erm*	
HB25411	*htrB*::*kan yhdK*::*erm*	
HB25412	*glpG*::*kan yhdK*::*erm*	
HB25413	*sipS*::*kan yhdK*::*erm*	
HB25414	*sipT*::*kan yhdK*::*erm*	
HB25415	*htpX*::*kan yhdK*::*erm*	
HB25416	*htrAB yhdK*::*erm*	
HB25417	*cssR yhdK*::*erm*	
HB25418	*cssS yhdK*::*erm*	
HB25419	*prsW*::*kan yhdK*::*erm*	
HB25420	*cssS htrB yhdK*::*erm*	
HB25421	*secDF yhdK*::*erm*	
HB25422	*sasA yhdK*::*erm*	
HB25423	*bshC yhdK*::*erm*	

HB25424	*msrA yhdK*::*erm*
HB25425	*nfrA yhdK*::*erm*
HB25426	*tpx yhdK*::*erm*
HB25427	*bshA yhdK*::*erm*
HB25428	*amyE*::$P_{spac(hy)}$-none *yhdK*::*erm*
HB25429	*amyE*::$P_{spac(hy)}$-*htrA yhdK*::*erm*
HB25430	*amyE*::$P_{spac(hy)}$-*htrB yhdK*::*erm*
HB25431	*sacA*::P_{htrA}-*lux yhdK*::*erm*
HB25432	*sacA*::P_{htrA}-*lux yidC1*^{Q140K} *yhdK*::*erm*

1. Rojas-Tapias DF & Helmann JD (2018) Induction of the Spx regulon by cell wall stress reveals novel regulatory mechanisms in Bacillus subtilis. *Mol Microbiol* 107(5):659-674.
2. Koo BM*, et al.* (2017) Construction and Analysis of Two Genome-Scale Deletion Libraries for *Bacillus subtilis*. *Cell Syst* 4(3):291-305 e297.
3. Chiba S, Lamsa A, & Pogliano K (2009) A ribosome-nascent chain sensor of membrane protein biogenesis in *Bacillus subtilis*. *EMBO J* 28(22):3461-3475.
