# Supplementary material for: Mutations of the Bacillus subtilis YidC1 (SpoIIIJ) insertase alleviate stress associated with σM-dependent membrane protein overproduction
Source: PLoS Genet. 2019 Oct 18;15(10):e1008263. doi: 10.1371/journal.pgen.1008263 (PMC6827917; doi:10.1371/journal.pgen.1008263)
Supplement: S6 Table — (PDF) [file pgen.1008263.s012.pdf]

Table S6. Primers used in this study

Number	Name	Sequence
1295	kan-F	CAGCGAACCATTTGAGGTGATAGG
1296	kan-R	CGATACAAATTCCTCGTAGGCGCTCG
6580	sigM-F-NotI	ATCGGCGGCCGCGCACTATCTTTTGCGGCCAT
6581	sigM-R-HindIII	ATCGAAGCTTTGGTCGCTCATTTCCCCATT
6582	yhdL-up-F	GCCGTTTTCGTTGCGAGAAT
6583	yhdL-up-R	CGCCGACATTCGCTGATTTTTCCTGGTCGCTCATTTCCC
6584	yhdL-mid-F	GGGAAATGAGCGACCAGGAAAAATCAGCGAATGTCGGCG
6585	yhdL-mid-R	CCTATCACCTCAAATGGTTCGCTGTCCGAAAACCGGTATAACGAAA
6586	yhdL-down-F	CGAGCGCCTACGAGGAATTTGTATCGAGATACGAATTTACAGTTTGGCT
6587	yhdL-down-R	ACGAATCGGGCAATCATGTG
6588	chr-sigM-seq-F	CCATTGTGCCACTCCTTCAC
6589	chr-sigM-seq-R	TGCAGTCATTTCCTGGTCGC
6590	pAX01-check-F	GGGGGAAATGACAAATGGTCC
6591	pAX01-check-R	ACGAAAGGGCCTCGTGATAC
6599	Pxyl-yhdL-F-BamHI	ATCGGGATCCTAGAGGGGAGAAAAGGCAATGATGAATGAAGAATTTAAAAAGC
6600	Pxyl-yhdL-R-SacII	ATCGCCGCGGTCCAGCCGAATACATTGTG
6693	pAX01-erm-cm-up-F	GCCGCACTCTTCCTTTTTCAA
6694	pAX01-erm-cm-up-R	CTTGATAATAAGGGTAACTATTGCCTTTGGTTGAGTACTTTTTCACTCG
6695	pAX01-erm-cm-down-F	GGGTAACTAGCCTCGCCGGTCCACGCTGGGGGAGGAAATAATTCTATGAGTCGC
6696	pAX01-erm-cm-down-R	TCGGCATTTTTGCATGGAGC
6759	yhdL-check-F	ACGCTGGGAAGCTACCTCTA
6760	yhdL-check-R	TCTGCTTTTGCGGTCGTTTG
6808	PsigM-F-EcoRI	AGCTGAATTCGCCGTTTGCATGTAATGTG
6809	PsigM-R-PstI	AGCTCTGCAGCAGTAAGTCTTCAGCAAGATGC
6814	pBs1ClacZ(lux)-check-F	AAAGGATTTGAGCGTAGCGA
6815	pBs1ClacZ-check-R	TTGGGTAACGCCAGGGTTTT
6816	pBs3Clux-check-R	GAGAGTCCTCCTGTCGACCT
6861	cssS-check-F	CCGCGAGGTCTATGACGAAA
6862	cssS-check-R	AGCTCAAGCGAAAGGGTGAA

6878	pBs3CLux-Cat-up-F	TGTTTGACAGCTTATCATCGGC
6879	pBs3CLux-Cat-Erm-up-R	CCTATCACCTCAAATGGTTCGCTGACTAAAAGTCGTTTGTTGGTTCA
6880	pBs3CLux-Cat-Erm-down-F	CGAGCGCCTACGAGGAATTTGTATCGTCAGATAGGCCTAATGACTGGC
6881	pBs3CLux-Cat-down-R	TTCGCGATCCGAAGTATGGG
7348	pJOE8999-check-F	CCTTTTTGCGTGTGATGCGA
7349	pJOE8999-check-R	GTCAGCTAGGAGGTGACTGA
7426	Delta-Pam-rodA-up-F	AAGGCCAACGAGGCCTCTGCTGAACACAGTCACTT
7427	Delta-Pm-rodA-up-R	CGCTTTTTCAGCTACACGAAATGCGATAATGTGTTATGTTCCC
7428	Delta-Pm-rodA-down-F	GGGAACATAACACATTATCGCATTTCGTGTAGCTGAAAAAGCG
7429	Delta-Pm-rodA-down-R	AAGGCCTTATTGGCCCTCATTTGAAGCAGACACCC
7430	ProdA-deltaPm-gRNA-F	TACGCGTTTTTTAACAAATTCTAT
7431	ProdA-deltaPm-gRNA-R	AAACATAGAATTTGTTAAAAAACG
7815	yidC1-seq-F	ACGGGAGATAACTACGGGCT
7816	yidC1-seq-R	GCTTCATCGACATTTCGCCC
7866	yidC1-gRNA-F	TACGATCCAATTAAAATCGGCATC
7867	yidC1-gRNA-R	AAACGATGCCGATTTTAATTGGAT
7868	yidC1-up-F	AAGGCCAACGAGGCCTATTGCCAGAAAACCGGCGA
7869	yidC1-up-R	TCGCATGATAGAATCCAATTAAGATAGGCATCTTGATCAAAATCGGGAAACATCCC
7870	yidC1-down-F	GGGATGTTTCCCGATTTTGATCAAGATGCCTATCTTAATTGGATTCTATCATGCGA
7871	yidC1-down-R	AAGGCCTTATTGGCCATCAGACTTCCCGGCAATGG
8120	yidC2-check-F	TCCTGCTCTAACGGCAATCG
8121	yidC2-check-R	CTTTTTGCACGGGGTTGCTT
8246	yidC1-PY79-to-168-down-F	CATTGGCGGGATGTTTCCCGATCTTGATCCAGATGCCGATTTTAATTGGA
8247	yidC1-PY79-to-168-up-R	AAAATCGGCATCTGGATCAAGATCGGGAAACATCCCGCCAATG
8248	yidC1-PY79-to-168-gRNA-F	TACGATCGGCATCTTGATCAAAAT
8249	yidC1-PY79-to-168-gRNA-R	AAACATTTTGATCAAGATGCCGAT
8250	jag-check-F	TGCGATCATGAGAACCCAGG
8251	jag-check-R	AGTTTGGTTCGAAGTGGAAGA
8264	PsigM-F-HindIII	ATCGAAGCTTGCCGTTTGCATGTAATGTG
8265	PsigM-R-BamHI	ATCGGGATCCCAGTAAGTCTTCAGCAAGATGC
8266	pDG1663-check-F	CCAACATGACGAATCCCTCC
8267	pDG1663-check-R	TAAGTTGGGTAACGCCAGGG

8276	YidC2-SpeI-F	ATCGACTAGTACCGCATTTATAAAAAGGAGGAGAA
8277	YidC2-BamHI-R	ATCGGGATCCTCAGCCATGATAAACAAGACT
8278	yidC1-R73A-gRNA-F	TACGAATTAATAAACGAATTAAAA
8279	yidC1-R73A-gRNA-R	AAACTTTTAATTCGTTTATTAATT
8280	YidC1-R73A-repair-up-R	GCTGCTTAATCATCAGCGGTAAAATTAATAATGCAATTAAAATGGTAACTAGAATAATTGAAAGC
8281	YidC1-R73A-repair-down-F	GCTTTCAATTATTCTAGTTACCATTTTAATTGCATTATTAATTTTACCGCTGATGATTAAGCAGC
8282	yidC1-SpeI-F	ATCGACTAGTAGATTAATTATAGGAGGAAATGTTGT
8283	yidC1-BamHI-R	ATCGGGATCCAGCAGTCACATTCCTCACTTTT
8341	Ec-YidC-seq-F	CCACGCCTGACGAGAAGTAT
8342	Ec-YidC-seq-R	AGTTTGAACAGCGGCTGAGA
8343	Ec-YidC-HindIII-F	ATCGAAGCTTTAAGGAGGACTAACGATGGATTCGCAACGCA
8344	Ec-YidC-XbaI-R	ATCGTCTAGAAGCGAAAACTCACCGAATCAGGA
8345	YidC2-HindIII-F	ATCGAAGCTTACCGCATTTATAAAAAGGAGGAGAA
8346	YidC2-XbaI-R	ATCGTCTAGATCAGCCATGATAAACAAGACT
8347	yidC1-XmaI-F	ATCGCCCGGGAGATTAATTATAGGAGGAAATGTTGT
8348	YidC1-XbaI-R	ATCGTCTAGAAGCAGTCACATTCCTCACTTTT
8349	jag-XbaI-R	ATCGTCTAGAAGTTTGGTTCGAAGTGGAAGA
8352	pDG1663-up-F	TTGGGTAACGCCAGGGTTTT
8353	pDG1663-spec-up-R	CGTTACGTTATTAGCGAGCCAGTCTGGTTGAGTACTTTTTCATTCGTT
8354	pDG1663-spec-down-F	CAATAAACCCTTGCCCTCGCTACGTCAAGCAATGAAACACGCCA
8355	pDG1663-down-R	ACCGCTGTGTTCGGATCTTT
8364	Ec-YidC-Q429K-up-R	AGGAAGATTGGCATCTTGATCAGCAGCGGGAAGCA
8365	Ec-YidC-Q429K-down-F	CTTCCCGCTGCTGATCAAGATGCCAATCTTCCTGGCGT
8371	htrA-check-F	CTGTTCCATCGACTCAGTCCT
8372	htrA-check-R	CGCAGATCATACCCAGTCCC
8373	htrB-check-F	AGAGCGAGGAAGATGTAGGA
8374	htrB-check-R	TCGGCCTGGCTGAAGAAAAT
8375	htpX-check-F	CGCACCATATCGGTTCGAGA
8376	htpX-check-R	AACGGCCACAGTAACTGCAA
8377	sipT-check-F	AGTATCGTGATCGGTGCTGT
8378	sipT-check-R	AAGCGCGGAAAAGAGAACAAA
8379	sipS-check-F	AGGCATGATGTGGGTAGAAGA

8380	sipS-check-R	ACGATGCATAACGGGAATATGT
8381	prsW-check-F	GGCATATCGCAGCGGAAATC
8382	prsW-check-R	TTCAAGCCTCCTACTGCAAA
8383	cssR-check-F	TCCTCGCTCTTTTTCTCTTCCT
8384	cssR-check-R	AGGCGGTACTCTGTCAGAAC
8385	cssR-RT-F	AGGCGAAAGATCCTGACGTG
8386	cssR-RT-R	CTCACGAGAGTATGGATGCCC
8389	cssS-int-F	TTATCGGGACGATTTGGCCT
8390	cssS-int-R	CTCTGATGACCATGACCGGC
8399	htrA-HindIII-F	ATCGAAGCTTTAAGGAGGGAACATGATGGATAACTATCGTGA
8400	htrA-XmaI-R	ATCGCCCGGGTTTACGGCCTGAGGCATTAT
8401	htrB-XmaI-F	ATCGCCCGGGTAAGGAGGTAAGAACATGGATTATCGACGTGA
8402	htrB-XbaI-R	ATCGTCTAGAATGCTTTCCTCTTATTTAGGGTAACA
8403	PhtrA-XbaI-F	ATCGTCTAGATCAACAGCTGTCTAGCGAT
8404	PhtrA-PstI-R	ATCGCTGCAGTCTCTATTTTCACATGTCTATTTATATTGA
8442	htrA-int-F	GCAACAAGCACCTCCTCTGA
8443	htrA-int-R	GTCCACGCCGCTTACAATTC
8444	htrB-int-F	ACGAACGCATCAAACATCGC
8445	htrB-int-R	TGTCAATCATCTGCACGCCT
8652	bshC-check-F	TGCTGGTTGACGTCATTGGA
8653	bshC-check-R	TGCTCACCTAGGCCTTCTCT
8654	msrA-check-F	TGAATTCATTCCGCGACAGC
8655	msrA-check-R	CCGTGCCGTTATTTTGCGTT
8656	nfrA-check-F	GTCAGCTATGGGGGAAGCTC
8657	nfrA-check-R	GTTTCTGCATTGCTGCCCTC
8658	tpx-check-F	TTGTCGGATCGACAGGCATC
8659	tpx-check-R	CACCGCACCTACATGGTCTT
8676	cssS-int-F	TTATCGGGACGATTTGGCCT
8677	cssS-int-R	CTCTGATGACCATGACCGGC
8681	yidC1-spc-2-R	CTCTTGCCAGTCACGTTACGTTATTAGCTGCAGCAGTCACATTCCTCA
8682	yidC1-spc-3-F	TGAGGAATGTGACTGCTGCAGCTAATAACGTAACGTGACTGGCAAGAG
8686	yidC1-MUT-2-F	ATTACCGCTGATGATTAAGCAGCT

8687	yidC1-MUT-3-F	TATTGGATTCTATCATGCGATCAT
8688	yidC1-MUT-4-F	GTAACTTGTTTATGATTGCGCAAACT
8689	yidC1-MUT-1-R	AGCTGCTTAATCATCAGCGGTAATMTTAATAATYGTMTTAAAATGGTAACTAGAATAATT
8690	yidC1-MUT-2-R	ATGATCGCATGATAGAATCCAATAMGAATCGGCATTTKGATCAAAATCGGGAAACATCCC
8691	yidC1-MUT-3-R	AGTTTGCGCAATCATAAACAAGTTACSAACTACCCRATAAAGAGAAAGAGCCGCCGGGAA
8700	dinB-RT-F	CGGCTGGATTGAAGTGTTTT
8701	dinB-RT-R	TGTTTTCATGATTCCGCTTG
8702	lexA-RT-F	GTTCCTCCAGACGAGCATGT
8703	lexA-RT-R	CAAGCGAATGTGGGTATCCT
8712	recA-RT-F	GTTCGGCAAAGGTTCCATTA
8713	recA-RT-R	GCCAATTCCCAGTGCTGTAT
8722	topA-RT-F	CAGCTGACCCCGACAGAGAA
8723	topA-RT-R	ATACGTCTCGCTTGCTGTGC
8726	gyrA-RT-F	GGCGGCCATGCGTTATACAG
8727	gyrA-RT-R	GCCATACCTACCGCAATGCC
8728	gyrB-RT-F	GTGTAGGTGCGTCGGTCGTA
8729	gyrB-RT-R	GCTAATTCACGCACGCGGTT
8736	secG-RT-F	GTGCCGGATTATCTGGTGCG
8737	secG-RT-R	AAGACTGCCAGCACTACCGT
8759	secDF-check-F	GAAGAGGGCCAGGAAGCATT
8760	secDF-check-R	CATTTGCCTTGCTTCAGCGT
8766	thrC-up-1-F	TATGCATCAGGTCGGCTGTC
8767	thrC-yidC1-1-R	TAAGAAAGCAGTGGTGATGCCAGGATTGACGCCTTCCGTTT
8768	thrC-yidC1-2-F	AAACGGAAGGCGTCAATCCTGGCATCACCACTGCTTTCTTA
8769	spec-thrC-3-R	GCAAACACGCCTTCTACACGGCAAGGGTTTATTGTTTTCTAAAATCTG
8770	spec-thrC-down-4-F	CAGATTTTAGAAAACAATAAACCCTTGCCGTGTAGAAGGCGTGTTTGC
8771	thrC-down-4-R	TTCCCCCTCTCCCAAACTGA
8772	thrC-yidC1-check-F	GATCAAACACCGGCGCTAAC
8773	thrC-yidC1-check-R	CAACTCCTGATCCAAACATGTAAGT
8774	yidC1-MUT-seq-F	GATAGTCCGCATTTCTGGGA
8775	yidC1-MUT-seq-R	TCTTGCCAGTCACGTTACGT
